# The case for a universal hepatitis C vaccine to achieve hepatitis C elimination

**DOI:** 10.1186/s12916-019-1411-9

**Published:** 2019-09-18

**Authors:** Nick Scott, David P. Wilson, Alexander J. Thompson, Eleanor Barnes, Manal El-Sayed, Adele Schwartz Benzaken, Heidi E. Drummer, Margaret E. Hellard

**Affiliations:** 1 Disease Elimination Program, Burnet Institute, Melbourne, Australia; 2 Department of Epidemiology and Preventive Medicine, Monash University, Clayton, Australia; 3 Department of Medicine, The University of Melbourne, Parkville, Australia; 4 Department of Gastroenterology, St Vincent’s Hospital Melbourne, Melbourne, Australia; 5 Nuffield Department of Medicine, University of Oxford, Oxford, UK; 6 NIHR Oxford Biomedical Research Centre, Oxford University NHS Trust, Oxford, UK; 7 Department of Paediatrics, Ain Shams University, Cairo, Egypt; 8 Global Medical Director for Aids Health Care Foundation, Brasília, DF Brazil; 9 Department of Microbiology, Monash University, Clayton, Australia; 10 Peter Doherty Institute for Infection and Immunity, The University of Melbourne, Parkville, Australia; 11 Department of Infectious Diseases, The Alfred Hospital and Monash University, Melbourne, Australia

**Keywords:** Elimination, Hepatitis C, Mathematical model, Vaccine

## Abstract

**Background:**

The introduction of highly effective direct-acting antiviral (DAA) therapy for hepatitis C has led to calls to eliminate it as a public health threat through treatment-as-prevention. Recent studies suggest it is possible to develop a vaccine to prevent hepatitis C. Using a mathematical model, we examined the potential impact of a hepatitis C vaccine on the feasibility and cost of achieving the global WHO elimination target of an 80% reduction in incidence by 2030 in the era of DAA treatment.

**Methods:**

The model was calibrated to 167 countries and included two population groups (people who inject drugs (PWID) and the general community), features of the care cascade, and the coverage of health systems to deliver services. Projections were made for 2018–2030.

**Results:**

The optimal incidence reduction strategy was to implement test and treat programmes among PWID, and in settings with high levels of community transmission undertake screening and treatment of the general population. With a vaccine available, the optimal strategy was to include vaccination within test and treat programmes, in addition to vaccinating adolescents in settings with high levels of community transmission. Of the 167 countries modelled, between 0 and 48 could achieve an 80% reduction in incidence without a vaccine. This increased to 15–113 countries if a 75% efficacious vaccine with a 10-year duration of protection were available. If a vaccination course cost US$200, vaccine use reduced the cost of elimination for 66 countries (40%) by an aggregate of US$7.4 (US$6.6–8.2) billion. For a US$50 per course vaccine, this increased to a US$9.8 (US$8.7–10.8) billion cost reduction across 78 countries (47%).

**Conclusions:**

These findings strongly support the case for hepatitis C vaccine development as an urgent public health need, to ensure hepatitis C elimination is achievable and at substantially reduced costs for a majority of countries.

**Electronic supplementary material:**

The online version of this article (10.1186/s12916-019-1411-9) contains supplementary material, which is available to authorized users.

## Background

There are vast differences in the epidemiology and burden of hepatitis C across the globe that have significant implications for meeting the World Health Organization (WHO) 2030 hepatitis C elimination targets. A consequence is that responses to hepatitis C must be tailored to particular settings; major considerations include whether the epidemic is concentrated (e.g. among key populations such as people who inject drugs [PWID]) or generalised, the transmission mechanisms (e.g. syringe sharing versus iatrogenic transmission in healthcare settings), and the capacity of the health system to respond (e.g. infrastructure and human resources).

The majority of high-income countries have concentrated hepatitis C epidemics with transmission predominantly occurring among PWID [1–3]. In settings where the prevalence of hepatitis C among PWID is less than approximately 50%, mathematical models show that treatment-as-prevention can be effective at reducing incidence as long as treatment scale-up is rapid, has sufficiently high coverage, and is supported by harm reduction to minimise re-infection and prevent epidemic rebound [4]. Many high-income countries have well-established health systems that are equipped to do this, for example Australia, Canada, and a number of European countries [5–8]. However, if the prevalence of hepatitis C among PWID is extremely high (greater than approximately 75%, such as Fiji, Indonesia, Iran, Italy, Malaysia, Mexico, and Pakistan [9]), it is unlikely that even three monthly testing of PWID would be sufficient to achieve major reductions in incidence [4]. Settings with high prevalence among PWID are therefore likely to need significant additional prevention measures to achieve hepatitis C elimination.

Many low- and middle-income countries have generalised epidemics with disease driven by iatrogenic transmission in official and unofficial health care and through other mechanisms such as tattooing and barbershop practices, or they have mixed epidemics where there is generalised transmission in addition to transmission among PWID. The prospects of reducing hepatitis C incidence through treatment-as-prevention in these countries is low due to limited financial resources and infrastructure to support rapid testing and treatment scale-up, multiple sources of transmission, and minimal existing prevention interventions.

Vaccines for hepatitis C have been in pre-clinical development since the discovery of hepatitis C virus almost 30 years ago. Two candidates have reached phase I clinical trials, with one reaching phase II testing (see ClinicalTrials.gov NCT01436357) [10]. While the outcome of the phase II clinical trial of a vaccine designed to generate cellular immunity (released in 2019 [11]) did not show efficacy, there remains optimism about the possibility of developing a vaccine for the prevention of hepatitis C within the next 5–10 years. However, vigorous research and development of hepatitis C vaccine candidates and the immunological requirements for natural protective immunity, as well as overall commercial interest in vaccine development, has been dampened by the discovery of direct acting antiviral therapies (DAAs), highly effective treatments for hepatitis C. The global spotlight is currently focussed on the major immediate gains being achieved through treatment. While this is justifiable in the short-term, reflecting on achievements and lessons learnt in other disease areas makes it hard to imagine a vaccine not playing a central role in long-term hepatitis C control strategies for the majority of the world.

In this study, we used a mathematical model to project hepatitis C incidence reduction following the implementation of a variety of test and treat elimination strategies in 167 countries. The strategies included different testing frequencies for PWID and the implementation of general population screening programmes. For each country, we determined which approach could achieve the WHO elimination target of an 80% reduction in hepatitis C incidence by 2030 in the most cost-effective way (as measured by cost per case averted). Where this target was not achievable, we determined the non-dominated scenario with the greatest 2030 incidence reduction. The analysis was performed with and without a theoretical prophylactic hepatitis C vaccine, to determine if additional impact and/or cost savings could be achieved if a vaccine were available, and hence the role and relative importance that a future vaccine has in achieving hepatitis C elimination.

## Methods

### Model description

**Fig. 1:**
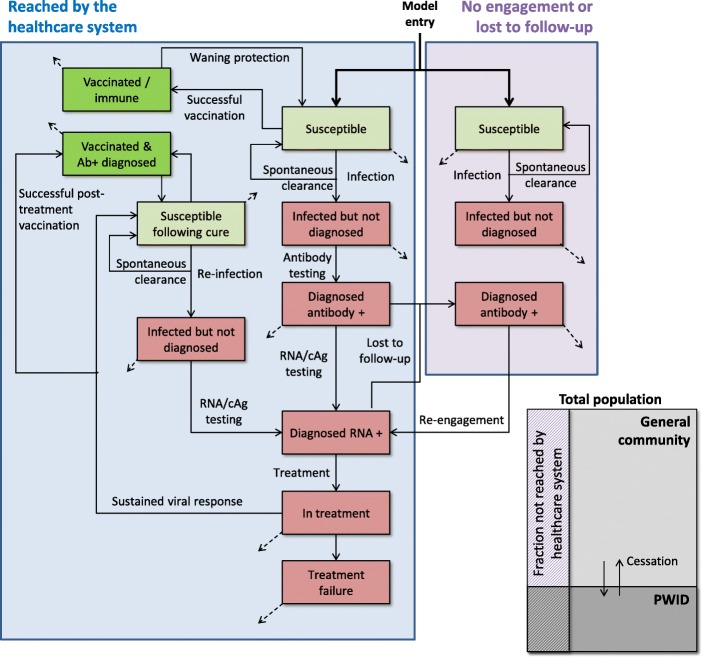
Model schematic

We used a dynamic compartmental model of hepatitis C transmission (Fig. 1). The model included two population groups: PWID and the “general community” (the general community as the 15–64-year-old population, as a proxy for non-PWID at risk of hepatitis C infection in settings with generalised or mixed epidemics).

Individuals from either population group could exit the model due to mortality at an average all-cause mortality rate, with PWID having an additional injecting-specific mortality rate. The total combined population size was held constant by the entry of susceptible individuals to the general community. After an average length of injecting career, PWID were assumed to cease injecting and move to the general community, with the total PWID population size maintained by recruitment from the general community.

People in the model were classified as susceptible (uninfected), vaccinated and immune (either through prophylactic vaccination or vaccination post-SVR12), or infected and classified as either undiagnosed, diagnosed antibody positive, diagnosed RNA/cAg positive, in treatment, or treatment failure. All cured individuals were assumed to be susceptible to reinfection and were moved to an alternate susceptible compartment to indicate that they would require future RNA/cAg testing rather than antibody testing due to known past exposure to hepatitis C. Susceptible individuals could become infected according to the current hepatitis C prevalence within their population group, whether or not they were covered by harm reduction (PWID population group only), and a population group-specific calibration constant that allows the model to be adapted to the hepatitis C prevalence in the given setting. For concentrated epidemic settings, the infection constant in the general community was set to zero. A proportion (26% [12]) of people were modelled to spontaneously clear and return to the susceptible compartment following infection.

Since not everyone is covered by private or public healthcare, the model includes a factor for the fraction of the population in each setting adequately covered with healthcare. Only the fraction who are adequately covered are able to be tested, treated or vaccinated, meaning that some people may never be diagnosed once they are infected. Prophylactic vaccination was modelled to be possible either as part of a testing programme (including following successful treatment completion), or as an age-based programme. In the age-based implementation, people were modelled to be vaccinated as they entered the model without the requirement of testing (i.e. turned 15 years). Vaccination was not modelled to occur for people who were currently chronically infected, including those for whom treatment failed. Cross-setting model parameters and setting-specific model parameters are shown in Table 2.

### Model calibration

The model was calibrated to 167 countries at the national level, based on population size estimates from UN Population Division [13] data (15–64 years), the estimated prevalence of injecting drug use [9] and the prevalence of hepatitis C among PWID [9] and the general population [14, 15] (Additional file 1: Appendix A). The models were calibrated by fitting the uptake rate of injecting drug use and the force of infection constants among PWID and the general community. Limited global datasets were available with hepatitis C prevalence estimates, meaning that the models were calibrated using flat epidemic curves (in the period before the strategies were implemented), to fit these estimates.

For each country, it was assumed that the fraction of the population adequately covered with healthcare (i.e. the reach of testing, treatment and vaccination programmes) was 80%, with the impact of 70% and 90% health system coverage also tested. For many countries, this would require substantive improvements in healthcare infrastructure and so should be considered to be the theoretical maximum attainable impact of hepatitis C elimination programmes. By assuming high coverage of testing and treatment programmes, we obtain conservative estimates for the additional impact of a vaccine.

As a means of highlighting some additional cross-setting differences and the impact of different efficacy vaccines, specific examples are presented for Australia, Brazil, China, Egypt, and the USA in Additional file 1: Appendix B.

In order to make the model outcomes relevant to sub-populations or sub-national geographical areas that may differ from national averages, independent models were calibrated for theoretical settings that had concentrated epidemics (25%, 50% or 75% hepatitis C prevalence among PWID), generalised epidemics (1%, 2%, 3%, 5%, 10%, 15%, 25% or 30% hepatitis C prevalence among the general community) and mixed epidemics (all combinations of hepatitis C prevalence among PWID and the general community). Results for these general settings are provided in Additional file 1: Appendix C.

### Scenarios considered

The interventions considered are described in Table 1 and include all combinations of (a) testing/treatment of PWID, (b) testing/treatment of the general community, (c) testing/treatment/vaccination of PWID, (d) testing/treatment/vaccination of the general community, and (e) an age-based vaccination programme for the general community. The model was projected between 2018 and 2030, and a 5-year period (2018–2023) was used to scale-up all interventions except age-based vaccination, which was modelled to begin immediately.

**Table 1:** Scenarios considered. Each setting was run with all combinations of the listed interventions and their variants

Intervention	Variants of intervention	Model implementation and assumptions
Testing/treatment among PWID	No testing or two yearly, annual, and six monthly testing	Testing coverage was modelled to be 80%, with 70% and 90% coverage used to derive uncertainty bounds. The programme assumed that following a positive antibody test, 80% of PWID were retained in care to have an RNA/cAg test within 3 months, and once diagnosed RNA/cAg + PWID would commence DAA treatment after an average of 60 days (with 95% success). Retention in care, time between follow-up tests and time to commence treatment were tested in the sensitivity analysis.
Testing/treatment among the general community	No testing or testing to result in the entire infected population being screened over a 12-year period (2018–2030)	This was implemented as 1/12th of people with hepatitis C in the general population being diagnosed every year between 2018 and 2030. Similar follow-up and treatment commencement assumptions to the testing interventions among PWID. Testing was only for people covered by the health system (80% of the population, with 70% and 90% used to derive uncertainty bounds).
Vaccination of PWID	No vaccination or a 75% efficacious vaccine with a 10-year duration of protection.	It was assumed that the vaccine was delivered with a test and treat strategy (frequency of testing being scenario dependent). Susceptible PWID were vaccinated after testing and infected PWID were treated and then vaccinated after SVR. The vaccine efficacy and duration of protection were tested in the sensitivity analysis. In particular, a scenario where the vaccine is only half as efficacious for people after successful treatment.
Combined testing and vaccination of the general community	No vaccination or a 75% efficacious vaccine with a 10-year duration of protection.	It was assumed that the vaccine was delivered with the general community testing programme. Susceptible people were vaccinated after testing and infected people were treated and then vaccinated. The vaccine efficacy and duration of protection were tested in the sensitivity analysis.
Age-based vaccination of the general community	No vaccination or a 75% efficacious vaccine with a 10-year duration of protection.	People were assumed to receive a vaccination as they entered the model (i.e. turned 15 years old), as this is a common age for delivering adolescent vaccination programmes. The vaccine efficacy and duration of protection were tested in the sensitivity analysis.

### Harm reduction among PWID

For each country, incidence rates and prevalence among PWID are partially driven by existing harm reduction coverage. Our main analysis assumed no changes to prevention interventions among PWID; however, alternate scenarios were tested in the sensitivity analysis, where prevention among PWID was scaled up by 20%, 40%, and 60% over a 5-year period (2018–2023). This was implemented as a relative reduction in the force of infection for the fraction of PWID considered to be adequately covered by harm reduction (Table 2).

**Table 2:** Cross-setting and country-specific parameters

**Vaccine parameters**		
Efficacy	75%	Assumed; tested in sensitivity analysis
Average duration of protection	10 years	Assumed; tested in sensitivity analysis
**Costs**		
Antibody tests	US$1.1	WHO estimate
RNA tests	US$20	WHO estimate
Antibody test positivity rate among PWID	1/PWID prevalence	Assumes frequency-based testing for PWID
Antibody test positivity rate among the general population	2/general population prevalence	Assumes general population testing populations are slightly targeted.
Treatment	US$150	WHO estimate, assuming generic pricings are available. Tested in the sensitivity analysis
Vaccination	US$200	Assumed; Tested in sensitivity analysis
Cost discounting	3% per annum	WHO recommendation [16]
**Hepatitis C-related parameters**		
Relative reduction in infection risk when covered by harm reduction	79%	Turner et al. [17], combined needle and syringe and opioid substitution therapy programmes.
Spontaneous clearance	26%	Micallef et al. [12]
Treatment effectiveness	95%	[18–21]
**Country-specific parameters: for specific country estimates, see Additional file **1: **Appendix A—Table S1**		
Total population size	UN Population Division [13]; 15–64 years (2016).	
Proportion of the population who inject drugs	Degenhardt et al. [9]. For countries without estimates, WHO region values were applied [22].	
Additional injecting-related mortality	Mathers et al. systematic review [23] (0.0235 per year)	
Epidemic type	Individual countries were classified as concentrated or mixed: epidemics were classified as mixed if the country was not in a WHO high income classification AND the total number of people living with hepatitis C was > 5 times the total number of estimated hepatitis C-infected PWID. This classification was used as without modelling transmission among the general population in these settings, the model was unable to produce the correct number of people living with hepatitis C based on injecting drug use-related transmission alone. Countries with general community transmission according to the above definition were classified as mixed rather than generalised (at the national level), since their PWID populations had significantly higher hepatitis C prevalence than the general community, and so the epidemics were assumed to have a concentrated component.	
Prevalence among PWID	Degenhardt et al. [9] For countries without estimates, population-weighted averages were calculated for each WHO region and applied.	
Prevalence in general population	Blach et al. [14] and Gower et al. [15] For countries without estimates, population-weighted averages were calculated for each WHO region and applied.	
Healthcare system coverage	Assumed to be 80% (with 70% and 90% used to derive uncertainty bounds). This parameter defines the coverage of testing / vaccination that could be achieved, and is used to derive uncertainty bounds for outcomes.	
Harm reduction coverage	Assumes that the status-quo is maintained for each country, with harm reduction scale-up tested in the sensitivity analysis.	
Staffing cost per interaction (testing+/−vaccination and treatment)	Estimated based on 2 h of provider time for interaction and any laboratory work. Average salary calculated as the population-weighted per capita gross domestic product (GDP) [24]. Assumes providers work 7 h per day, 5 days per week and 45 weeks per year. Tested in sensitivity analysis.	

### Costs

A healthcare provider’s perspective was taken for costs (Table 2). The commodity cost of hepatitis C antibody tests, RNA tests, and treatment were based on generic pricings. Human resource costs to deliver testing and treatment services were estimated based on 2 h of provider time for each interaction, using per capita gross domestic product as a proxy for providers’ wages.

Hepatitis C testing efficiency for PWID was based on prevalence (i.e. with 50% prevalence, it would require on average two tests to obtain one positive, assuming testing guidelines are frequency-based), while for the general community testing was assumed to be conducted twice as well as random selection (i.e. in a setting with 1% general population prevalence, this implies that 50 tests would be required to find one positive case, and therefore that testing programmes can be slightly targeted to those with possible exposure).

A vaccine was modelled with a baseline cost of US$200 per course based on realistic pricing from other vaccines [25], with costs of US$0–500 per course tested to determine the threshold price where interventions that included a vaccine would dominate non-vaccine interventions (i.e. cost less and avert more cases). It is likely that a vaccination course will require up to three doses, meaning that this price must include commodity and delivery costs for all doses. All costs were discounted at 3% [16].

### Outcome measures: optimal WHO incidence reduction target strategy

An *optimal WHO incidence reduction target strategy* was determined for each country, with and without a vaccine available, as follows. First, all combinations of non-vaccine interventions in Table 1 were run, and the corresponding cumulative incidence (2018–2030), cumulative costs of testing and treatment (2018–2030), and relative reduction in incidence by 2030 were recorded. Scenarios were considered dominated if they cost more but prevented fewer cumulative cases than another scenario, and dominated scenarios were excluded from further consideration. Among the non-dominated scenarios, the optimal strategy was considered to be the one that achieved an 80% reduction in incidence by 2030 in the most cost-effective way (as measured by cost per incident case averted). Where no scenarios could achieve this level of incidence reduction, the non-dominated scenario with the greatest 2030 incidence reduction was selected. This process was repeated with a broader set of scenarios that included all vaccine and non-vaccine intervention options. Case study examples are presented for Australia, Brazil, China, Egypt, and the USA in Additional file 1: Appendix B—Figure S5-S9.

### Uncertainty bounds

A Monte Carlo probabilistic uncertainty analysis was undertaken, where 100 random input parameter sets were drawn and uncertainty in outputs were estimated as the central 95 percentiles of these runs (parameters for the force of infection constants, treatment efficacy, costs, rates of retention in care, and times to progress along the care cascade were randomly selected from their uncertainty ranges or +/− 10% using uniform probability distributions). However, uncertainty in the coverage of health service parameter (when it was varied between 70 and 90%) was more than the combined uncertainty from these parameters. As this is also the parameter with the most uncertainty and practical implications, in order to better reflect true uncertainty we reported wider uncertainty bounds on our outputs corresponding to 70–90% health service coverage.

### Sensitivity analyses

The model was run with alternate assumptions including a 90% or 50% efficacious vaccine rather than 75%, a vaccine being only half as efficacious if administered after successful treatment, no staffing costs or double the staffing costs associated with delivering testing (+/− vaccination) and treatment services, 5 or 100 years duration of protection from the vaccine rather than 10 years, an average of 90 days between Ab and RNA tests rather than 60 days, antibody testing and RNA testing occurring on the same day (to reflect a rapid point-of-care antibody test), an average of 60 days from diagnosis to treatment rather than 30 days, 70% retention in care following a positive antibody test rather than 80%, and half the test positivity rate among the general community (reflecting testing at random). These alternate assumptions were run on the theoretical settings in order to assess any dependence on epidemic type and initial prevalence, and the results are provided in Additional file 1: Appendix D—Table S6.

## Results

Without a vaccine, the optimal incidence reduction strategy was to implement test and treat programmes among PWID with setting-specific testing frequencies and to undertake screening and treatment of the general population in settings with high levels of community transmission. With a vaccine available, the optimal strategy was to include vaccination within these test and treat programmes, in addition to vaccinating adolescents in settings with high levels of community transmission (Additional file 1: Appendix E—Table S8). For 69 (41%) of the countries modelled, a vaccine reduced the testing frequency required among PWID.

**Fig. 2:**
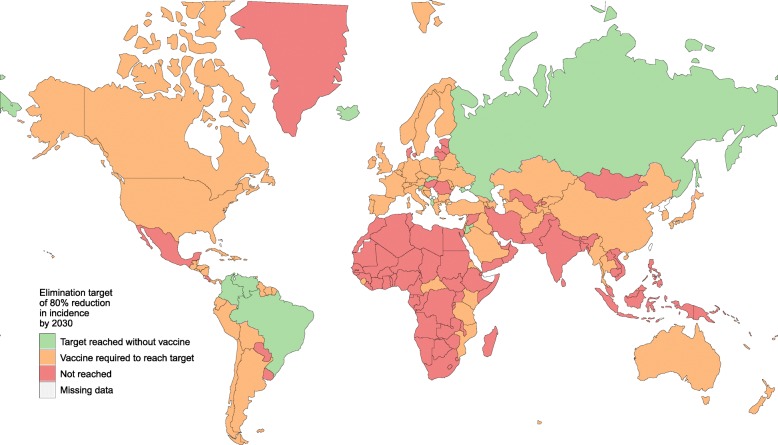
Countries where the WHO target of an 80% reduction in incidence by 2030 could be reached with or without a vaccine available. Countries are shown according to whether (a) there was a non-dominated scenario without a vaccine that reached the target (green), (b) the only non-dominated scenarios that reached the target required a vaccine (orange), or (c) there were no non-dominated scenarios that reached the target (red). Projections assume 80% coverage of testing, treatment and vaccination programmes, and a maximum testing frequency of six monthly among PWID

**Fig. 3:**
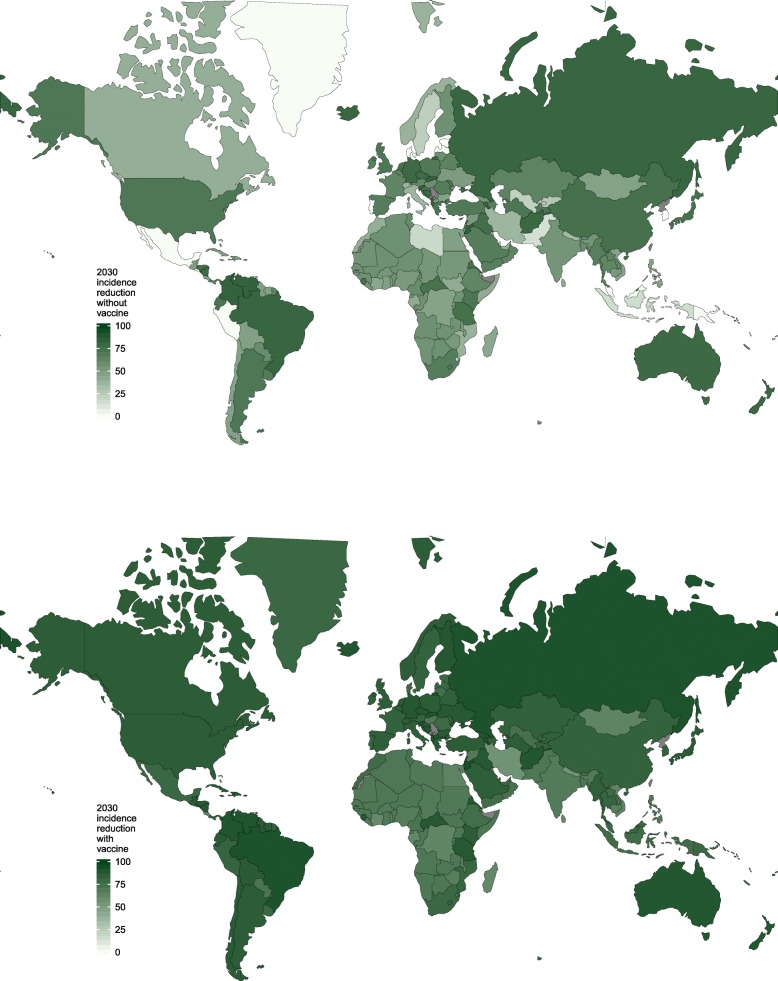
Relative reduction in incidence by 2030, projected for the optimal WHO incidence reduction target strategies without (top) and with (bottom) a vaccine available. Projections assume 80% coverage of testing, treatment and vaccination programmes, and a maximum testing frequency of six monthly among PWID

**Fig. 4:**
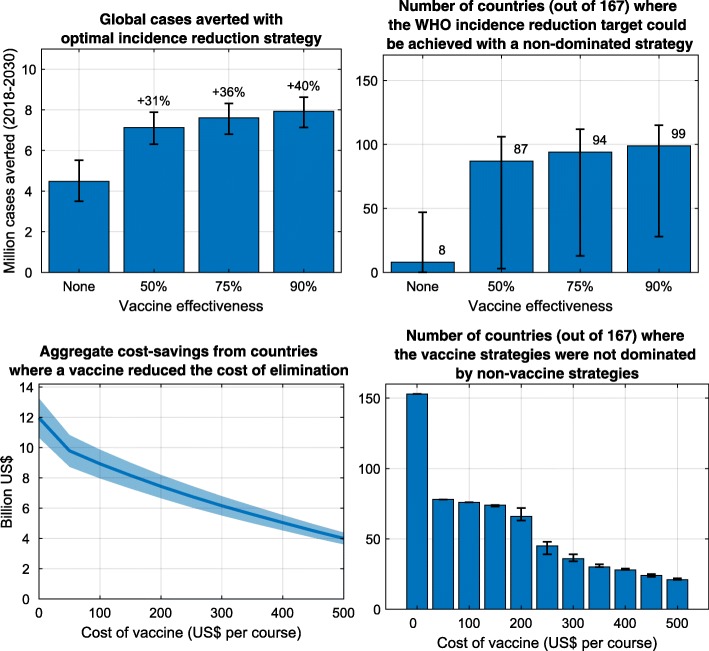
Estimated impact of a vaccine on the feasibility and cost of hepatitis C elimination. Total cases averted 2018–2030 using the optimal incidence reduction strategies without a vaccine, or with a vaccine that was 50%, 75%, or 90% efficacious (top left); and the number of countries where the WHO incidence reduction target could be achieved with a non-dominated strategy (top right). For different vaccine costs, the number of countries where a vaccine was a component of the optimal strategy (bottom right); and the total reduction in the cost of elimination if it were used in these countries. Uncertainty bounds represent scenarios with 70% and 90% population coverage of testing, treatment, and vaccination compared to a base of 80%

Of the 167 countries modelled, between 0 and 48 countries (0–29% of countries, depending on programme coverage ranging from 70 to 90%) could achieve an 80% reduction in incidence using testing and treatment alone (Additional file 1: Appendix E—Table S7). The median relative reduction in hepatitis C incidence in the optimal strategies without a vaccine was 55% (inter-quartile range [IQR] 46–70%) (Additional file 1: Appendix E—Table S7 and Fig. 3, top). With a 75% efficacious vaccine, between 15 and 113 countries (9–68%) could achieve the 80% incidence reduction target (Additional file 1: Appendix E—Table S7, Figs. 2 and 4). The median relative reduction in incidence in the optimal strategies with a vaccine available was 81% (IQR 73–86%) (Additional file 1: Appendix E—Table S7 and Fig. 3, bottom).

For countries with concentrated epidemics, the price point for a vaccine to reduce the total cost of the optimal strategy was a median US$247 (IQR US$204–442) per course (Additional file 1: Appendix E—Table S7). Below these threshold prices, the savings from less frequent testing and treatment requirements among PWID outweighed the additional cost of the vaccine. For countries with mixed epidemics, the price point for a vaccine to reduce the total cost of the optimal strategy was a median US$1.36 (IQR US$0.94–3.04) per course (Additional file 1: Appendix E—Table S7). The price point was much lower for settings with mixed epidemics because the vaccine needed to be delivered at much larger scale.

As the price per vaccination course decreased, the availability of a vaccine reduced the cost of the optimal strategy for an increasing number of countries. At US$200 per vaccination course, the optimal strategy with a vaccine was cheaper than the optimal strategy without a vaccine for 66 (40%) of the countries analysed, leading to an aggregate cost reduction of US$7.4 (US$6.6–8.2) billion across these countries compared to the optimal strategies without a vaccine available (Fig. 4). This increased to 78 (47%) of the countries analysed and an aggregate cost reduction of US$9.8 (US$8.7–10.8) billion for a US$50 per course vaccine.

For 14 countries (8%), the optimal non-vaccine strategy involved no testing and treatment programme among PWID. Common to these countries is a very high hepatitis C prevalence among PWID (> 80%). The extremely high prevalence in these settings meant that there were not many uninfected PWID, and so hepatitis C incidence was low. Scaling up treatment led to an increase in the number of susceptible PWID and a subsequent increase in incidence. Therefore, according to our modelled definition of optimal, the non-vaccination strategy with the lowest cumulative incidence in 2030 involved no intervention or cost, but also had no impact on the epidemic. This illustrates some of the challenges faced when aiming for elimination in high prevalence settings.

## Discussion

This study shows that vaccines can play an important role in the elimination of hepatitis C. We found that for the majority of settings, the WHO target of an 80% reduction in hepatitis C incidence by 2030 is unlikely to be achieved without additional prevention measures. Integrating a vaccine within hepatitis C testing and treatment programmes could greatly improve the feasibility and likelihood of reaching the WHO elimination incidence reduction target by directly reducing transmission and reducing the high-frequency testing burden among risk populations.

Without a vaccine or significant additional prevention measures, fewer than 29% of countries analysed were able to reduce hepatitis C incidence by 80% by 2030 in the model (Additional file 1: Appendix E—Table S7). With a vaccine available, this could increase to as many as 68% of countries, depending on programme coverage (Additional file 1: Appendix E—Table S7). More generally, when a vaccine was available the optimal incidence reduction strategy had approximately double the impact in generalised epidemic settings and approximately four times the impact in settings with a high prevalence among PWID (Additional file 1: Appendix C—Figure S11). This clearly demonstrates that while testing and treatment programmes have the potential to produce major gains towards hepatitis C elimination, they have limitations that will require additional intervention, and a hepatitis C vaccine is worth pursuing to fill this gap.

A vaccine also improved the feasibility of reaching the WHO incidence reduction target for many settings in the model, by reducing the testing burden. Consistent with previous work [4], settings with high prevalence among PWID required high-frequency testing programmes in order to reduce incidence. The optimal incidence reduction strategies for 86% of countries required six monthly testing of PWID (Additional file 1: Appendix E—Table S7). Such a high testing frequency would be burdensome to both individuals and healthcare systems meaning that for many countries this target may not be achievable through testing and treatment alone. With a vaccine available to reduce reinfection, testing requirements among PWID were reduced to either two yearly for 47 (28%) of the 167 countries or annually for 26 (16%) of the 167 countries (Additional file 1: Appendix E—Table S7), which is likely to be considerably more feasible.

For concentrated epidemic settings, which represent the majority of developed countries, a vaccine could significantly reduce the costs of hepatitis C elimination. When a vaccine was modelled at US$200 per dose, 66 countries were identified where it would reduce the cost of reaching the WHO incidence reduction target, with an aggregate cost saving of US$7.4 billion. This is because of (a) the reduced number of PWID requiring regular screening because of their vaccination status, (b) the reduced frequency of screening required, and (c) the reduced number of treatments required. Compared to achieving elimination with testing and treatment alone, the price point to save costs by including a vaccine was a median US$247 (IQR US$204–442) and US$1.36 (IQR US$0.94–3.04) per course in countries with concentrated and mixed epidemic settings, respectively. Therefore, a vaccine for hepatitis C must be cheap and simple to manufacture so that it is economically feasible to deliver at scale in low- and middle-income countries, with some costs potentially offset by profits in high-income countries. By way of comparison, the sale price of vaccines for other diseases varies greatly by pathogen and also by country [26]. For example, the Centre for Disease Control in the USA prices adult vaccines for hepatitis B at US$30.81; human papillomavirus at US$144.18; measles, mumps, and rubella at US$45.65; and varicella at US$77.57 [27]. However, in low- and middle-income countries, the hepatitis B vaccine can cost as little as US$0.16 per dose [26]. This study provides an estimation of the economic benefits of a vaccine in different settings and for a range of vaccine prices, which may be useful for vaccine developers, programme managers, and policy makers.

These results suggest that without additional prevention measures, the optimal global incidence reduction strategy for hepatitis C is to implement targeted test and treat programmes among those at high risk of hepatitis C infection and to implement hepatitis C vaccination through these programmes. It is estimated that only 20% of people with hepatitis C were diagnosed globally in 2015 [22], meaning that broad screening programmes will be required among risk populations in most settings to achieve elimination. This would provide an opportunity to achieve relatively high vaccination coverage, in particular among PWID. In addition, for settings with high rates of transmission in the general community, the model suggests that introduction of a vaccination programme for adolescents would provide additional benefit (Additional file 1: Appendix C—Table S4, generalised settings). It is therefore worth examining the potential to combine a hepatitis C vaccine with other vaccines targeting this age group such as human papillomavirus or hepatitis B.

Harm reduction for PWID has an important role to play in the hepatitis C elimination agenda. In sensitivity analyses, we tested the impact that scaling up prevention measures among PWID could have on the testing requirements, vaccination requirements, and costs of achieving the incidence reduction target. Consistent with previous modelling [7, 28], increasing prevention programmes was found to increase the impact of testing and treatment by reducing the need for retreatment (Additional file 1: Appendix D). Therefore, programmes such as needle and syringe distribution and opioid substitution therapy should be considered essential components of viral hepatitis strategies as recommended by the WHO [29].

The total cost of reaching the WHO hepatitis C elimination targets has been estimated at US$51 billion globally [30]. For many countries, the direct costs associated with hepatitis C are currently low, because there are no or limited services available to manage hepatitis C-related liver disease. As a result, the required outlay on hepatitis C testing and treatment programmes represents new costs with minimal immediate direct economic benefits. However, when the indirect economic benefits of hepatitis C elimination are considered, such as a larger and more productive workforce, this spending has been estimated to lead to a US$19 billion return on investment by 2030 [30]. The issue of cost is therefore largely one of affordability. This is relevant because in this study we have modelled the use of a vaccine alongside the scaling up of other programmes as a way to improve prevention and reduce hepatitis C transmission—only one component of hepatitis C elimination—and the vaccine was modelled to be delivered through testing and treatment programmes because they will also be required to reduce hepatitis C morbidity and mortality. Our estimate that a US$200 per course vaccine could reduce the cost of elimination by US$7.4 billion should therefore be interpreted as approximately a 15% reduction in the estimated total cost, should financing be obtained for hepatitis C elimination.

The total cost of developing a vaccine from discovery through to licensure varies widely but has been estimated at US$0.5–1.0 billion [31]. The results of the first phase II efficacy trial of a prophylactic hepatitis C vaccine designed to generate cellular immunity, released in 2019, showed no impact [11]. While much can be learned from this study, it is a reminder that a vaccine for hepatitis C will still require significant research and development investment. By comparison, billions of dollars have been invested in HIV vaccine development; in 2019, there were 39 ongoing HIV vaccine trials, with 28 past and current phase II trials [32]. For hepatitis C, the market size of a vaccine will be determined by who is to be vaccinated (target population), how often (life-long versus waning immunity with a need for revaccination) and which countries adopt a vaccination programme, with most profits derived in developed countries. However, a lack of data and a reliance on DAAs to underpin global elimination efforts is blocking interest in hepatitis C vaccine development. Like almost all vaccines, a public-private partnership is likely to be required to fund a future candidate, but to form such partnerships there must be a shift in global opinion towards one that supports the urgent development of a preventive vaccine.

The main limitations of this study relate to the hypothetical vaccine. Prophylactic vaccines for hepatitis C are currently undergoing clinical trials, and their final properties are yet to be determined. First, for our main analysis, we assumed a 75% efficacious vaccine was available with a 10-year duration of protection, which is conservative compared to what may be achievable. Second, we assumed that the vaccine had a single efficacy value (modelled as a population average across genotypes and age groups) and that the vaccine efficacy was the same in naive individuals as previously infected individuals. It remains to be determined if the immune system is fully restored after attaining DAA-mediated viral clearance and is responsive to hepatitis C vaccination. However, our sensitivity analysis suggests that vaccine efficacy post viral clearance does not change the optimal strategy and has a minor impact on cost. Third, we used a single average cost for a vaccine course that is likely to require multiple doses. Some of the costs of delivering these doses can be minimised by combining with existing interactions with healthcare providers; for example, in the scenarios being considered, the initial dose for adults is delivered with a testing interaction and so no additional staff costs would be required, and for adolescents much of the delivery costs could be avoided by inclusion with human papillomavirus or hepatitis B vaccine schedules. When a vaccine is available, it will also be important to consider the acceptability of the vaccine among all population groups and the potential for loss to follow-up between doses. The potential impact of these assumptions for our results is that (1) the average vaccine efficacy may be different than estimated (e.g. corresponding to the 50% efficacy scenarios in Fig. 4), (2) the cost of delivery may be higher than estimated (corresponding to the higher cost scenarios in Fig. 4), or (3) the achievable coverage may be reduced due to loss to follow-up between doses or people opting out of vaccination (corresponding to the lower bounds of error bars). In each of these cases, the model still projects that epidemiological and economic benefits can be gained through vaccine development.

Much of the cost-effectiveness of a future vaccine will be driven by currently unknown properties such as duration of protection, storage requirements, shelf life, number of doses required for protection, and cross-genotype efficacy. For example, if a vaccine were only effective for genotype 1, additional genotype testing may be required alongside our modelled “test (treat) and vaccinate” strategies which would greatly influence cost. If or when a vaccine does become available, follow-up studies are warranted to assess and compare the cost-effectiveness of different implementation strategies against various willingness-to-pay thresholds. Instead, the primary aim of this study was to generate evidence that a vaccine for hepatitis C has the potential to unlock health and economic benefits beyond what is achievable with DAAs alone. We modelled vaccine interventions to be scaled up between 2018 and 2023 and maintained until 2030. While this cannot happen as a vaccine is many years away, this time period is the window where DAA treatments are expected to have their greatest impact, with the model projecting that even in this period a vaccine would be a critical biomedical intervention. As more treatment uptake and epidemic data become available, new vaccine scenarios should be modelled based on the updated and more detailed epidemic curves of individual countries, in particular in the context of stabilised treatment.

## Conclusion

These findings strongly support the case for hepatitis C vaccine development as an urgent public health need, to ensure hepatitis C elimination is achievable and at substantially reduced costs for most countries.

## Additional file

Additional file 1: This file contains **Appendix A.** country specific data inputs (population sizes, prevalence of hepatitis C among PWID and the general community, and staff costs); **Appendix B.** specific country case studies; **Appendix C.** general settings (results from concentrated, generalised and mixed epidemic settings, with hepatitis C prevalence of 25/50/75% among PWID +/− 1/2/3/5/10/15/20/25/30% among the general community); **Appendix D.** sensitivity analysis (impact of varying harm reduction coverage, vaccine efficacy after treatment, staff costs, vaccine duration of protection, and assumptions about test positivity and progression through the cascade of care); and **Appendix E.** country specific results. (PDF 1989 kb)

## Data Availability

All data generated or analysed during this study are included in this published article and its supplement (Additional file 1).

## Abbreviations

cAg: Core antigen
DAA: Direct-acting antiviral
PWID: People who inject drugs
RNA: Ribonucleic acid
US$: United States dollar
WHO: World Health Organization
