# Supplementary material for: The case for a universal hepatitis C vaccine to achieve hepatitis C elimination
Source: BMC Med. 2019 Sep 18;17:175. doi: 10.1186/s12916-019-1411-9 (PMC6749704; doi:10.1186/s12916-019-1411-9)
Supplement: Supplementary file 1 — This file contains Appendix A. country specific data inputs (population sizes, prevalence of hepatitis C among PWID and the general community, and staff costs); Appendix B. specific country case studies; Appendix C. general settings (results from concentrated, generalised and mixed epidemic settings, with hepatitis C prevalence of 25/50/75% among PWID +/− 1/2/3/5/10/15/20/25/30% among the general community); Appendix D. sensitivity analysis (impact of varying harm reduction coverage, vaccine efficacy after treatment, staff costs, vaccine duration of protection, and assumptions about test positivity and progression through the cascade of care); and Appendix E. country specific results. (PDF 1989 kb) [file 12916_2019_1411_MOESM1_ESM.pdf]

Supplementary material: The case for a universal hepatitis C vaccine to achieve hepatitis C elimination

APPENDIX A: COUNTRY SPECIFIC MODEL INPUTS

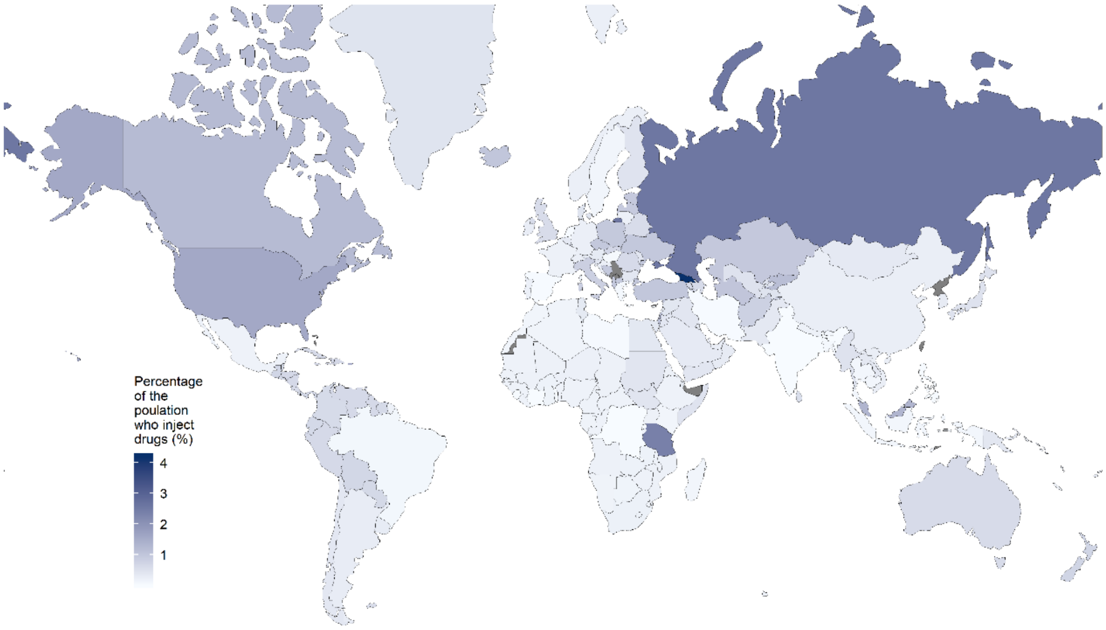

Figure S1: Proportion of the population who inject drugs. Based on Degenhardt et al.[1] For countries without estimates, population-weighted averages were calculated for each WHO region and applied.

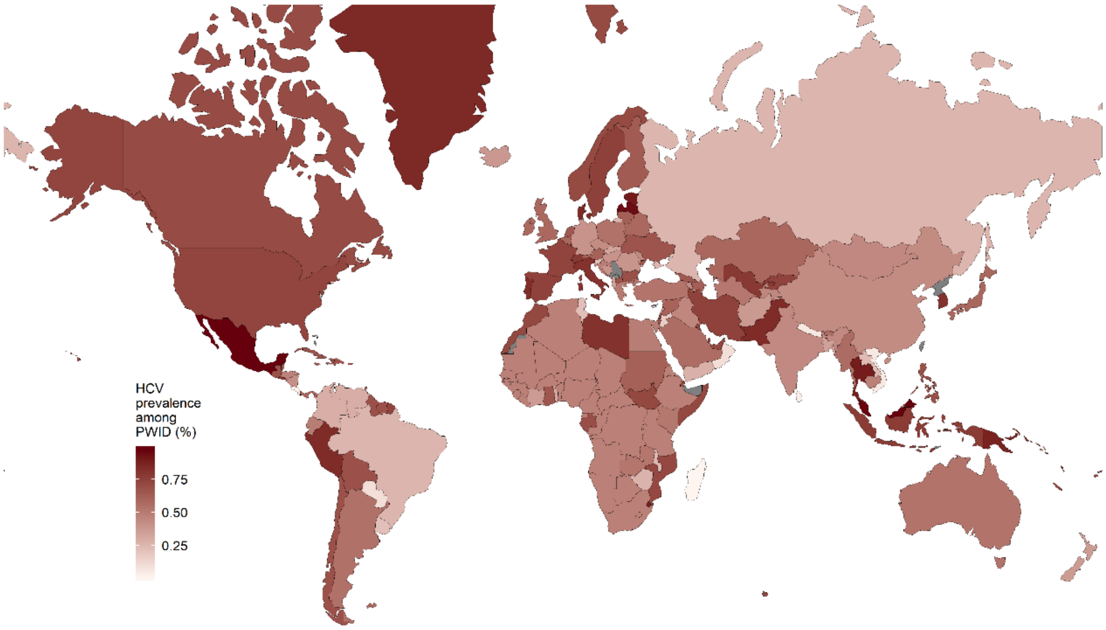

Figure S2: hepatitis C prevalence among people who inject drugs used for model input. Based on Degenhardt et al.[1] For countries without estimates, population-weighted averages were calculated for each WHO region and applied. Grey countries are missing data.

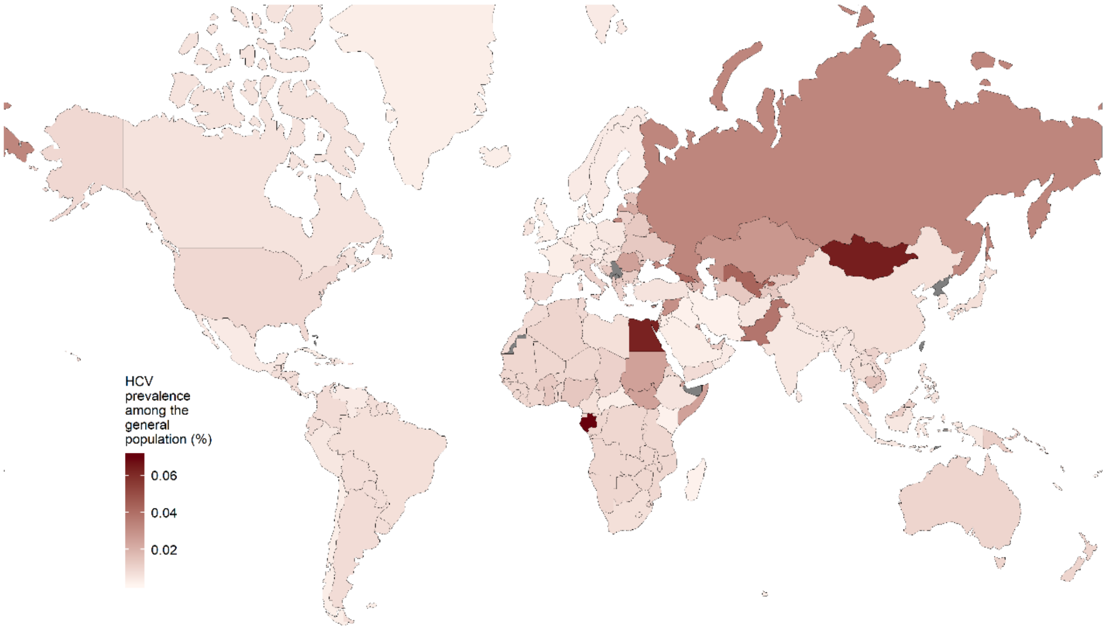

Figure S3: hepatitis C prevalence among the general population used for model input. Based on Blach et al.[2] and Gower et al.[3] For countries without estimates, population-weighted averages were calculated for each WHO region and applied.

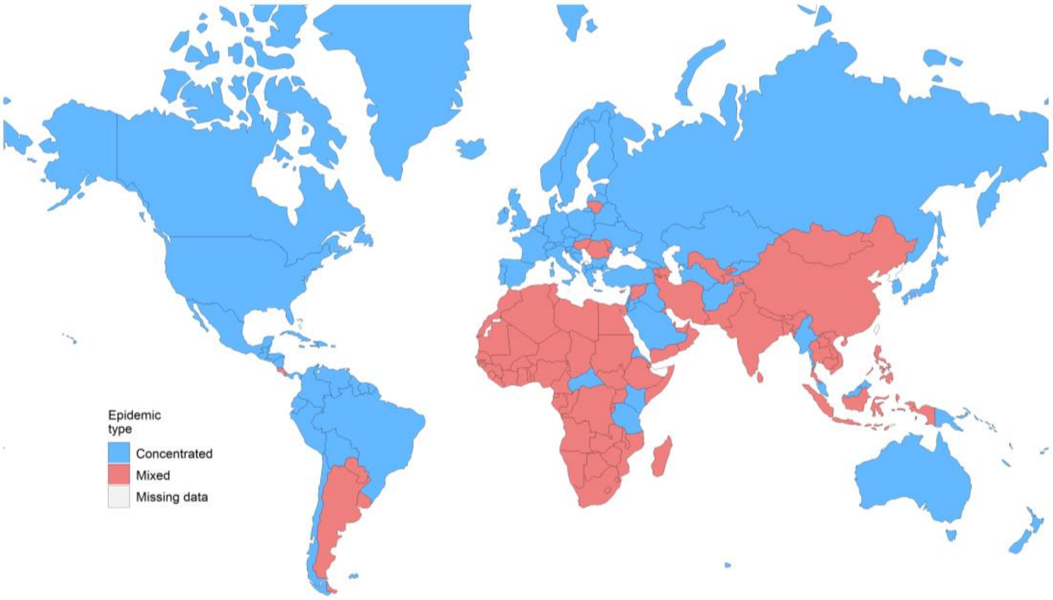

Figure S4: Epidemic type classification used for model input. Epidemics were classified as mixed, meaning that infection was possible among the general population as well as among PWID, if the country was not in a WHO high income classification AND the total number of people living with hepatitis C was >5 times the total number of estimated hepatitis C-infected PWID. This was done as without transmission among the general population, the model was unable to produce the correct number of people living with hepatitis C based on injecting drug use-related transmission alone.

Table S1: Country specific model inputs.

Country	Epidemic type[a]	Adult population (2016) [b]	Percent of the population who inject drugs[c]	Total number of PWID[d]	hepatitis C prevalence among PWID[e]	hepatitis C prevalence among the general population[f]	Staff costs per testing, treatment, or vaccination interaction[g]
Afghanistan	concentrated	18,575,258	0.80%	148,602	38%	0.50%	$0.71
Albania	concentrated	1,997,271	0.66%*	13,182	34%	1.34%*	$5.24
Algeria	mixed	26,383,347	0.10%*	26,383	48%*	1.00%	$4.97
Angola	mixed	14,605,793	0.10%*	14,606	48%*	0.95%*	$4.20
Antigua and Barbuda	concentrated	69,717	0.42%*	293	68%*	0.75%*	$18.36
Argentina	mixed	28,009,966	0.29%	81,229	55%	0.80%	$15.80
Armenia	mixed	2,018,577	0.62%	12,515	43%	1.34%*	$4.59
Australia^^	concentrated	15,942,528	0.60%	95,655	40%	0.74%	$63.18
Austria	concentrated	5,839,284	0.32%	18,686	61%	0.20%	$56.84
Azerbaijan	mixed	6,926,250	0.61%	42,250	62%	1.90%	$4.93
Bahrain	mixed	1,102,591	0.23%*	2,536	72%*	1.20%	$28.67
Bangladesh	mixed	107,583,339	0.07%	75,308	34%	0.51%*	$1.73
Belarus	concentrated	6,554,281	0.59%	38,670	58%	1.34%*	$6.34
Belgium	concentrated	7,324,673	0.35%	25,636	58%	0.60%	$52.41
Belize	concentrated	235,740	0.42%*	990	68%*	0.75%*	$6.03

Benin	mixed	5,859,997	0.10%*	5,860	48%*	0.95%*	$1.00
Bhutan	mixed	544,687	0.10%	558	57%*	0.51%*	$3.52
Bolivia	concentrated	6,683,948	0.42%*	28,073	68%*	0.75%*	$3.94
Bosnia and Herzegovina	concentrated	2,449,320	0.66%*	16,166	40%	1.34%*	$6.11
Botswana	mixed	1,452,622	0.10%*	1,453	48%*	0.95%*	$8.79
Brazil	concentrated	144,560,447	0.10%	144,560	26%	0.70%	$10.98
Brunei	concentrated	305,929	0.23%*	704	48%*	0.74%*	$34.21
Bulgaria	concentrated	4,663,920	0.38%	17,723	69%	1.20%	$9.48
Burkina Faso	mixed	9,733,933	0.10%*	9,734	48%*	1.30%	$0.80
Burundi	mixed	5,535,519	0.10%*	5,536	48%*	1.00%	$0.36
Cambodia	mixed	10,143,144	0.11%	11,157	48%*	1.60%	$1.61
Cameroon	mixed	12,641,093	0.10%*	12,641	48%*	0.70%	$1.75
Canada	concentrated	24,462,794	1.22%	298,446	71%	0.60%	$53.78
Central African Republic	concentrated	2,427,789	0.10%*	2,428	48%*	0.30%	$0.49
Chad	mixed	7,247,070	0.10%*	7,247	48%*	1.10%	$0.84
Chile	concentrated	12,304,561	0.38%	46,757	68%*	0.30%	$17.51
China	mixed	995,072,896	0.25%	2,487,682	43%	0.70%	$10.32
Colombia	concentrated	33,463,378	0.42%*	140,546	29%	0.80%	$7.37
Comoros	mixed	454,278	0.10%*	454	48%*	0.95%*	$0.98
Costa Rica	mixed	3,345,218	0.42%*	14,050	2%	0.75%*	$15.02
Croatia	concentrated	2,754,710	0.23%	6,336	50%	0.60%	$15.43
Cuba	concentrated	7,979,569	0.42%*	33,514	68%*	0.30%	$12.70*
Cyprus	mixed	819,142	0.08%	655	18%	2.49%*	$29.89
Czechia	concentrated	6,999,761	0.97%	67,625	43%	0.40%	$23.47
Democratic Republic of the Congo	mixed	39,867,115	0.02%	7,874	48%*	0.95%*	$2.27*
Denmark	concentrated	3,664,484	0.45%	16,490	85%	0.30%	$68.04
Djibouti	mixed	605,638	0.23%*	1,393	72%*	2.49%*	$7.26*
Dominican Republic	concentrated	6,767,761	0.42%*	28,425	68%*	0.60%	$8.54
Ecuador	concentrated	10,550,807	0.42%*	44,313	49%	0.75%*	$7.64
Egypt^^	mixed	58,789,880	0.23%*	135,217	35%	4.46%	$4.42
El Salvador	concentrated	4,067,975	0.42%*	17,085	79%	0.75%*	$5.36
Equatorial Guinea	mixed	730,225	0.10%*	730	48%*	0.95%*	$11.11
Eritrea	concentrated	2,406,710	0.10%*	2,407	48%*	0.95%*	$2.27*
Estonia	concentrated	850,705	0.94%	7,997	91%	1.40%	$22.52
Ethiopia	mixed	56,725,456	0.10%*	56,725	48%*	0.60%	$0.90
Fiji	concentrated	587,548	0.23%*	1,351	74%	0.10%	$6.65
Finland	concentrated	3,453,483	0.46%	15,886	64%	0.40%	$55.15
France^^	concentrated	41,793,933	0.20%	83,588	60%	0.24%	$46.80
Gabon	mixed	1,181,086	0.10%*	1,181	69%	7.00%	$9.12
Gambia	mixed	1,062,851	0.10%*	1,063	48%*	0.80%	$0.60
Georgia	concentrated	2,466,709	4.19%	103,355	65%	4.20%	$4.91
Germany	concentrated	54,145,785	0.24%	129,950	40%	0.30%	$53.54
Ghana	mixed	16,341,478	0.10%*	16,341	66%	1.40%	$1.92

Greece	concentrated	7,050,669	0.07%	4,935	50%	1.10%	$22.72
Guatemala	concentrated	9,907,744	0.42%*	41,613	68%*	0.75%*	$5.27
Guinea	mixed	6,748,593	0.10%*	6,749	48%*	0.95%*	$0.84
Guinea-Bissau	mixed	1,006,798	0.10%*	1,007	48%*	0.95%*	$0.81
Guyana	concentrated	505,827	0.42%*	2,124	68%*	0.75%*	$5.75
Haiti	concentrated	6,719,916	0.42%*	28,224	68%*	0.75%*	$0.94
Honduras	concentrated	5,756,968	0.42%*	24,179	47%	0.75%*	$3.00
Hungary	mixed	6,637,582	0.06%	3,983	40%	0.50%	$16.28
Iceland^^	concentrated	220,576	0.66%*	1,456	38%#	0.2%	$76.86
India	mixed	873,908,707	0.04%*	349,563	44%	0.50%	$2.17
Indonesia	mixed	175,328,142	0.11%	192,861	77%	0.50%	$4.53
Iran	mixed	57,086,320	0.28%	159,842	75%	0.20%	$6.63
Iraq	concentrated	20,962,705	0.23%*	48,214	45%	0.20%	$5.85
Ireland	concentrated	3,073,960	0.27%	8,300	58%	0.60%	$81.49
Israel	concentrated	5,180,967	0.66%*	34,194	68%	1.20%	$47.21
Italy^^	concentrated	38,609,106	0.83%	320,456	72%	0.98%	$38.94
Ivory Coast	mixed	12,917,333	0.01%	1,292	37%	0.95%*	$1.95
Jamaica	concentrated	1,945,801	0.42%*	8,172	68%*	0.75%*	$6.20
Japan^^	concentrated	76,831,284	0.47%	361,107	49%	0.58%	$49.49
Jordan	concentrated	5,714,481	0.23%*	13,143	16%	0.30%	$5.19
Kazakhstan	concentrated	11,701,081	0.96%	112,330	59%	2.80%	$9.80
Kenya	concentrated	27,372,406	0.12%	32,847	51%	0.20%	$1.85
Kuwait	mixed	3,113,472	0.23%*	7,161	72%*	2.49%*	$34.74
Kyrgyzstan	concentrated	3,898,697	0.74%	28,850	74%	1.34%*	$1.37
Laos	mixed	4,242,601	0.23%*	9,758	23%	0.74%*	$2.97
Latvia	concentrated	1,278,276	0.92%	11,760	95%	2.20%	$17.87
Lebanon	concentrated	4,091,304	0.23%*	9,410	41%	0.20%	$10.49
Lesotho	mixed	1,322,124	0.10%*	1,322	48%*	0.95%*	$1.32
Liberia	mixed	2,530,636	0.10%*	2,531	48%*	0.95%*	$0.58
Libya	mixed	4,230,347	0.05%	2,115	81%	0.70%	$31.98*
Lithuania	mixed	1,906,357	0.22%	4,194	62%	1.10%	$18.94
Luxembourg	concentrated	404,123	0.57%	2,304	81%	0.90%	$127.92
Macedonia	concentrated	1,464,085	0.66%*	9,663	67%	1.34%*	$6.65
Madagascar	mixed	13,898,297	0.12%	16,678	1%	0.20%	$0.51
Malawi	mixed	9,539,262	0.10%*	9,539	25%	0.95%*	$0.38
Malaysia	concentrated	21,613,466	1.33%	287,459	97%	1.20%	$12.07
Mali	mixed	8,932,211	0.10%*	8,932	48%*	0.95%*	$0.99
Mauritania	mixed	2,442,002	0.10%*	2,442	48%*	0.95%*	$1.40
Mauritius	concentrated	892,649	0.78%	6,963	48%*	0.95%*	$12.23
Mexico	concentrated	84,512,801	0.18%	152,123	97%	0.40%	$10.42
Moldova	mixed	2,626,915	0.40%	10,508	43%	1.34%*	$2.41
Mongolia	mixed	2,020,751	0.23%*	4,648	36%	5.15%	$4.69
Morocco	mixed	23,237,448	0.13%	30,209	72%*	0.80%	$3.67
Mozambique	mixed	14,943,954	0.20%	29,888	72%	0.95%*	$0.49
Myanmar	concentrated	35,494,275	0.48%	170,373	57%*	0.51%*	$1.52
Namibia	mixed	1,477,758	0.10%*	1,478	48%*	0.95%*	$5.61

Nepal	mixed	18,166,451	0.20%	36,333	6%	0.51%*	$0.93
Netherlands^^	concentrated	11,076,563	0.03%	3,323	49%	0.08%	$57.95
New Zealand	concentrated	3,059,011	0.73%	22,331	37%	1.00%	$50.05
Nicaragua	concentrated	4,012,544	0.42%*	16,853	42%	0.75%*	$2.73
Niger	mixed	9,761,597	0.10%*	9,762	48%*	0.95%*	$0.46
Nigeria	mixed	98,882,303	0.10%*	98,882	48%*	1.40%	$2.76
Norway	concentrated	3,433,905	0.24%	8,241	71%	0.40%	$89.99
Oman	mixed	3,346,874	0.23%*	7,698	10%	0.40%	$19.03
Pakistan	mixed	117,113,479	0.37%	433,320	84%	3.80%	$1.83
Panama	concentrated	2,607,394	0.42%*	10,951	59%	0.30%	$17.37
Papua New Guinea	concentrated	4,851,119	0.23%*	11,158	88%	1.20%	$3.17
Paraguay	mixed	4,309,857	0.42%*	18,101	10%	0.75%*	$5.18
Peru	concentrated	20,768,436	0.42%*	87,227	84%	0.50%	$7.68
Philippines	mixed	65,451,034	0.04%	26,180	69%	0.60%	$3.75
Poland	concentrated	26,202,640	0.66%*	172,937	54%	0.50%	$15.76
Portugal	concentrated	6,713,450	0.22%	14,770	83%	0.80%	$25.23
Qatar	mixed	2,182,534	0.23%*	5,020	78%	1.60%	$75.33
Republic of the Congo	mixed	2,779,937	0.10%*	2,780	48%*	0.95%*	$12.09
Romania	mixed	13,251,748	0.62%	82,161	39%	2.50%	$11.11
Russia	concentrated	99,477,057	1.78%	1,770,692	26%	3.30%	$11.11
Rwanda	mixed	6,750,468	0.03%	2,025	48%*	0.95%*	$0.89
Saudi Arabia	concentrated	23,013,048	0.23%*	52,930	56%	0.30%	$25.43
Senegal	mixed	8,323,176	0.10%*	8,323	48%*	0.95%*	$1.21
Singapore	concentrated	4,063,707	0.23%*	9,347	48%*	0.74%*	$67.25
Sierra Leone	mixed	4,072,319	0.04%	1,629	48%	0.95%*	$0.64
Slovakia	concentrated	3,809,944	0.49%	18,669	33%	0.60%	$20.99
Slovenia	concentrated	1,376,715	0.42%	5,782	22%	0.30%	$27.49
Solomon Islands	concentrated	343,603	0.23%*	790	48%*	0.74%*	$2.55
Somalia	mixed	7,266,202	0.23%*	16,712	72%*	2.49%*	$0.55
South Africa	mixed	36,753,491	0.21%	77,182	48%*	0.70%	$6.70
South Korea	concentrated	37,364,822	0.23%*	85,939	82%	0.50%	$34.97
South Sudan	mixed	6,685,064	0.23%*	15,376	72%*	2.49%*	$2.27*
Spain^^	concentrated	30,705,410	0.03%	9,212	56%	0.60%	$33.80
Sri Lanka	mixed	13,997,712	0.01%	1,400	3%	0.51%*	$4.87
Sudan	mixed	21,900,668	0.23%*	50,372	61%	2.49%*	$3.07
Suriname	concentrated	371,347	0.42%*	1,560	68%*	0.75%*	$7.46
Swaziland	mixed	798,701	0.10%*	799	89%	0.95%*	$3.52
Sweden	concentrated	6,228,380	0.13%	8,097	75%	0.40%	$65.83
Switzerland^^	concentrated	5,604,694	0.24%	13,451	72%	0.92%	$101.44
Syria	mixed	10,773,905	0.23%*	24,780	61%	3.00%	$7.26*
Tajikistan	mixed	5,367,478	0.45%	24,154	45%	1.34%*	$1.01
Timor-Leste	mixed	669,094	0.01%	67	57%*	0.51%*	$1.78
Thailand	mixed	49,164,792	0.11%	54,081	90%	0.70%	$7.51
Togo	mixed	4,210,790	0.06%	2,526	31%	0.95%*	$0.73

Tonga	concentrated	61,991	0.23%*	143	48%*	0.74%*	$4.76
Trinidad and Tobago	concentrated	949,398	0.42%*	3,987	68%*	0.75%*	$20.37
Tunisia	mixed	7,790,140	0.23%*	17,917	22%	0.90%	$4.68
Turkey	concentrated	53,061,615	0.66%*	350,207	53%	0.60%	$13.79
Turkmenistan	concentrated	3,681,434	0.66%*	24,297	52%	1.34%*	$8.11
Uganda	mixed	20,678,765	0.10%*	20,679	48%*	0.95%*	$0.74
Ukraine	concentrated	30,875,921	0.97%	299,496	67%	1.34%*	$2.78
United Arab Emirates	mixed	7,881,775	0.23%*	18,128	72%*	1.30%	$47.77
United Kingdom^^	concentrated	42,001,358	0.59%	247,808	53%	0.27%	$51.32
United Republic of Tanzania	concentrated	28,790,340	1.24%	357,000	48%*	0.95%*	$1.11
United States of America	concentrated	213,071,223	1.04%	2,215,941	53%	0.90%	$73.19
Uruguay	mixed	2,211,820	0.30%	6,635	22%	0.75%*	$19.33
Uzbekistan	mixed	21,558,308	0.47%	101,324	78%	4.30%	$2.68
Vanuatu	concentrated	161,124	0.23%*	371	85%	0.74%*	$3.63
Venezuela	concentrated	20,731,567	0.42%*	87,073	30%	0.40%	$12.70*
Vietnam	mixed	66,197,535	0.25%	165,494	6%	1.10%	$2.76
Yemen	mixed	15,681,743	0.23%*	36,068	28%	0.80%	$1.26
Zambia	mixed	8,696,624	0.10%*	8,697	52%	0.95%*	$1.61
Zimbabwe	mixed	9,012,715	0.10%*	9,013	27%	0.95%*	$1.31

* WHO regional estimate.

^^ In 2018 these countries were identified as the 12 that were on track to achieve elimination by 2030[4]. Therefore updated prevalence estimates were generated for PWID and the general community by subtracting the 2016 and 2017 treatment numbers[4] from the estimated number of people with hepatitis C (assuming PWID were equally likely to be treated as members of the general community).

[#] Scott et al. 2018[5]; pre-elimination program estimate.

[a] Epidemics were classified as generalised, meaning that infection was possible among the general population as well as among PWID, if the country was not in a WHO high income classification AND the total number of people living with hepatitis C was >5 times the total number of estimated hepatitis C-infected PWID. This was done as without transmission among the general population, the model was unable to produce the correct number of people living with hepatitis C based on injecting drug use-related transmission alone.

[b] UN Population Division[6]; 15-64 years (2016).

[c] Degenhardt et al.[1]. For countries without estimates, WHO region values were applied[7].

[d] Estimated based on prevalence of injecting drug use.

[e] Degenhardt et al.[1] For countries without estimates, population-weighted averages were calculated for each WHO region and applied.

[f] Blach et al.[2] and Gower et al.[3] For countries without estimates, population-weighted averages were calculated for each WHO region and applied.

[f]Average salary calculated as the per capita gross domestic product (GDP); from World Bank data[8]. Costs assume two hours of provider time for interaction and any laboratory work, and that providers work 7 hours per day, 5 days per week and 45 weeks per year.

APPENDIX B: SPECIFIC COUTNRY CASE STUDIES

Summary inputs

Table S2: Example model input parameters for five country case studies. Data for all 167 countries are in the supplement Table S1.

Country	Australia	Brazil	China	Egypt	United States of America
Total adult population	15,942,528	144,560,447	995,072,896	58,789,880	213,071,223
Percentage of the population who inject drugs	0.60%	0.1%[9]	0.25%	0.23%	1.04%
Total number of PWID	95,655	144,560	2,487,682	135,217	2,215,941
Epidemic type	concentrated	concentrated	mixed	mixed	concentrated
Prevalence among PWID	39.6%^^	26%[10,11]	43.1%	35.0%^^	53.1%
Prevalence among the general population	0.7%^^	0.7%[12]	0.7%	4.46%^^	0.9%
Staff costs associated with each interaction	US$63.18	US$10.98	US$10.32	US$4.42	US$73.19

^^ In 2018 these countries were identified as being among 12 that were on track to achieve elimination by 2030[4]. Therefore updated prevalence estimates were generated for PWID and the general community by subtracting the 2016 and 2017 treatment numbers[4] from the estimated number of people with hepatitis C (assuming PWID were equally likely to be treated as members of the general community).

Selecting the optimal strategy

The optimal WHO incidence reduction target strategies for each country with and without a vaccine available were chosen as follows, with five examples illustrated in Figures S5-S9.

First, all combinations of interventions without a vaccine were run and considered in the cost-effectiveness plane of total cost versus cumulative cases averted, 2018-2030. Specifically, test/treat programs for PWID [testing two-yearly, annually or six-monthly] with and without testing of the general community. Scenarios were considered dominated if they cost more but prevented fewer cumulative cases than another scenario, and dominated scenario were excluded from further consideration. Among the non-dominated scenario, the optimal WHO target strategy was considered to be the one that achieved an 80% reduction in incidence by 2030 in the most cost-effective way (as measured by cost per incident case averted). Where no scenario could achieve this level of incidence reduction, the non-dominated scenario with the greatest 2030 incidence reduction was selected.

This process was then repeated with a broader set of interventions that included the availability of a vaccine. Specifically, all combinations of test/treat programs for PWID [testing two-yearly, annually or six-monthly] with and without vaccination included; testing of the general community with and without vaccination included; and an adolescent vaccination program. Again the optimal WHO target strategy was considered to be the non-dominated scenario that was the most cost effective

for achieving an 80% reduction in incidence by 2030, or where this was not possible the non-dominated scenario that achieved the greatest 2030 incidence reduction.

As Australia, Brazil and USA were classified as having concentrated epidemics, the general community interventions are suppressed from those figures below. For Chine and Egypt, a log10 scale is used on the cost axis. In China, without a vaccine we see that the general community testing (squares) were only slightly more effective but much more expensive than PWID only testing (circles), and dominated by PWID only testing + vaccination (diamonds).

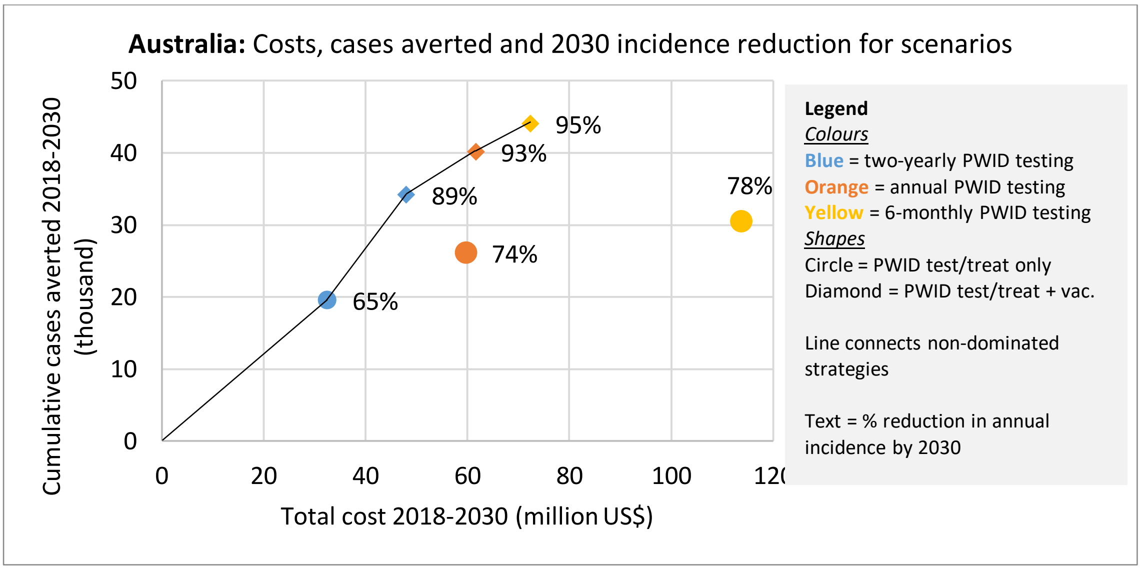

Figure S5: Selecting the optimal strategy for Australia. Without a vaccine (only circles considered), 6-monthly testing of PWID (yellow circle; 78% incidence reduction by 2030) was selected as the non-dominated optimal WHO target strategy. With a vaccine available, the most cost-effective way to reach an 80% reduction in incidence was a two-yearly test/treat/vaccinate program for PWID (blue diamond). Vaccine was assumed to have 75% efficacy, 10-year duration of protection and cost US$200/course.

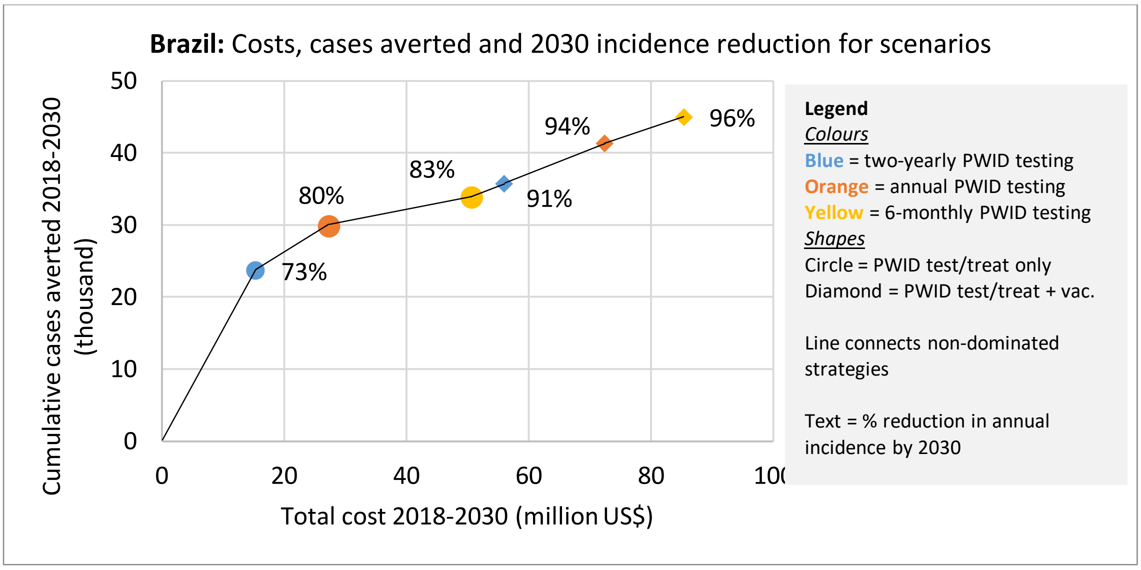

Figure S6: Selecting the optimal strategy for Brazil. With or without a vaccine, annual testing of PWID was the most cost-effective and non-dominated way to achieve an 80% reduction in incidence by 2030 (orange circle). Vaccine was assumed to have 75% efficacy, 10-year duration of protection and cost US$200/course.

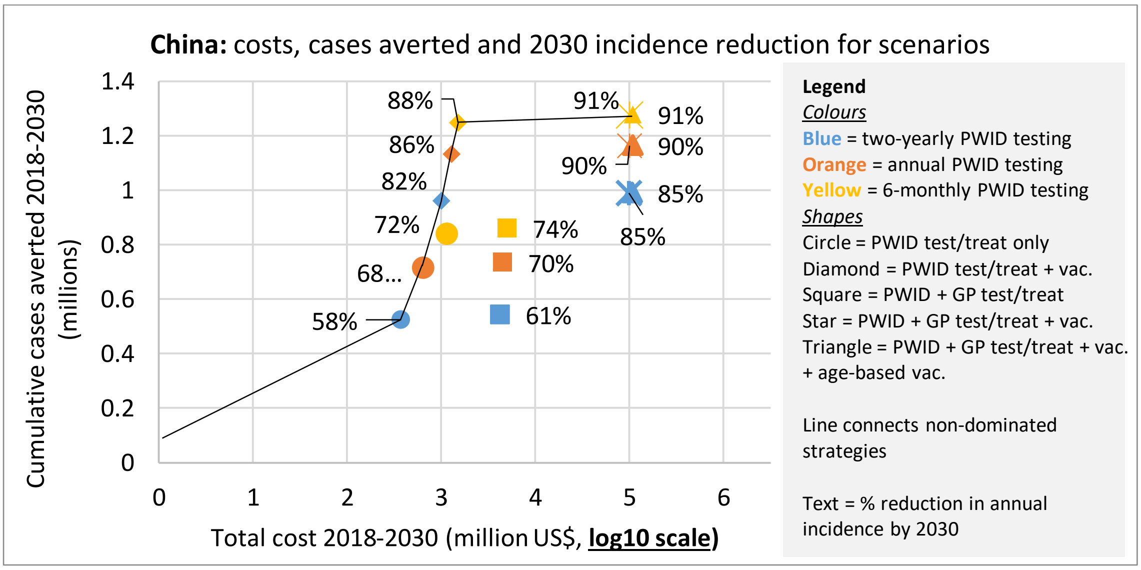

Figure S7: Selecting the optimal strategy for China. Without a vaccine (only circles and squares considered), 6-monthly testing of PWID + general population screening (yellow square; 74% reduction in incidence by 2030) was selected as the optimal WHO target strategy. With a vaccine available, the most cost-effective way to reach an 80% reduction in incidence was a two-yearly test/treat/vaccinate program for PWID (blue diamond). Vaccine was assumed to have 75% efficacy, 10-year duration of protection and cost US$200/course.

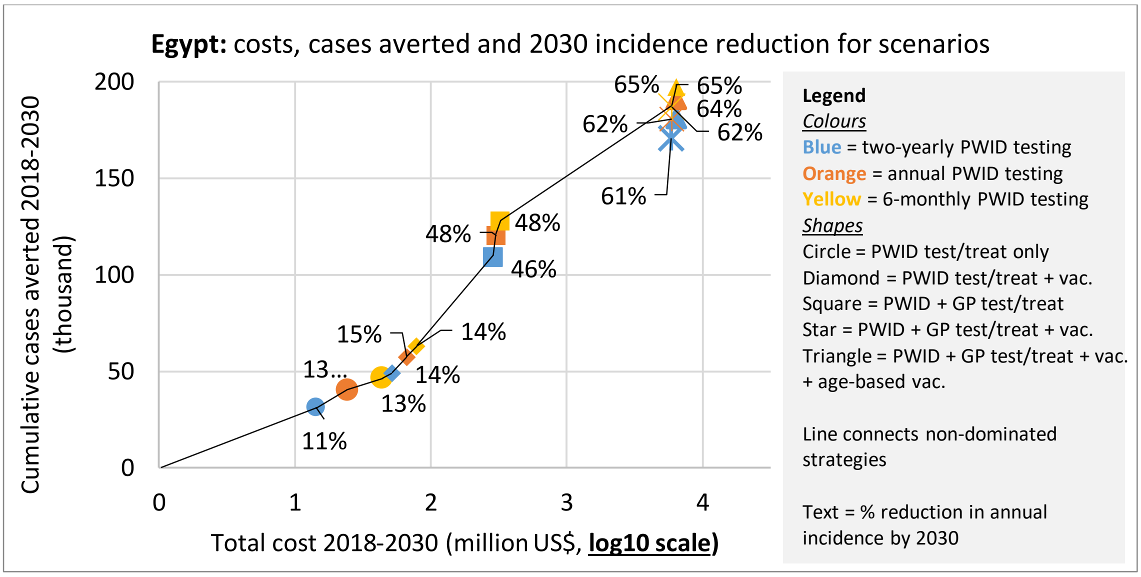

Figure S8: Selecting the optimal strategy for Egypt. Without a vaccine (only circles and squares considered), 6-monthly testing of PWID + general population screening (yellow square; 48% reduction in incidence by 2030) was selected as the optimal WHO target strategy. With a vaccine available, a 64% reduction in incidence by 2030 was the greatest non-dominated strategy, using two-yearly test/treat/vaccinate program for PWID and screening of the general population (without vaccination) (blue star). Vaccine was assumed to have 75% efficacy, 10-year duration of protection and cost US$200/course.

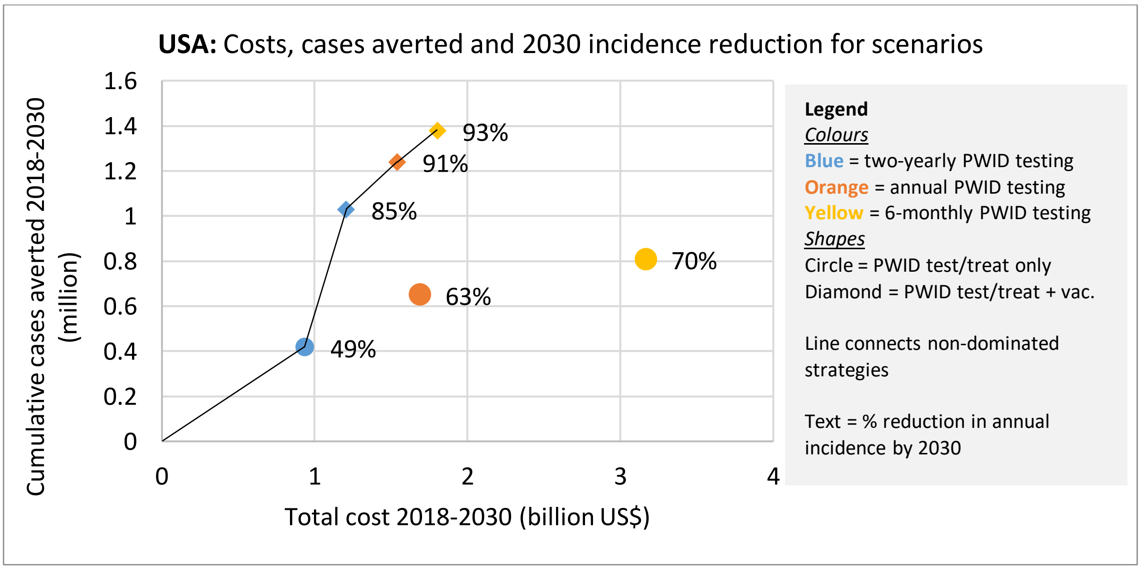

Figure S9: Selecting the optimal strategy for USA. Without a vaccine (only circles considered), 6-monthly testing of PWID (yellow circle; 70% incidence reduction by 2030) was selected as the optimal WHO target strategy. With a vaccine available, the most cost-effective way to reach an 80% reduction in incidence was a two-yearly test/treat/vaccinate program for PWID (blue diamond). Vaccine was assumed to have 75% efficacy, 10-year duration of protection and cost US$200/course.

Impact of a vaccine

Assuming 80% health care coverage, the availability of a 75% efficacious vaccine as a possible intervention for an elimination strategy led to an additional 16%, 13%, 20% and 25% of cases averted between 2018 and 2030 in Australia, China, Egypt and the USA, respectively, but no difference for Brazil as it did not form part of the optimal WHO target strategy in that setting (see Figure S6). With a 75% efficacious vaccine, testing requirements among PWID became more feasible, with testing requirements among PWID reduced from 6-monthly to two-yearly in Australia, China and the USA.

Availability of a vaccine could lead to different optimal strategies for reaching the WHO target. For China, the optimal strategy without a vaccine included screening of the general community, whereas the additional impact of a vaccine (even if only 50% efficacious) meant that it became possible to reduce incidence by 80% through targeted programs for PWID alone, which was considerably cheaper (Figure S10).

A US$200 per course 75% efficacious vaccine reduced the total costs of the optimal incidence reduction programs in Australia, China and the USA by US$66 million (58%), US$4.0 billon (80%) and US$2.0 billion (62%), respectively. For Egypt, unless the vaccine was under US$2.46 per course, the optimal strategy with a vaccine resulted in higher total costs, due to the large-scale vaccine delivery required through the age-based vaccination program and the general population screening program.

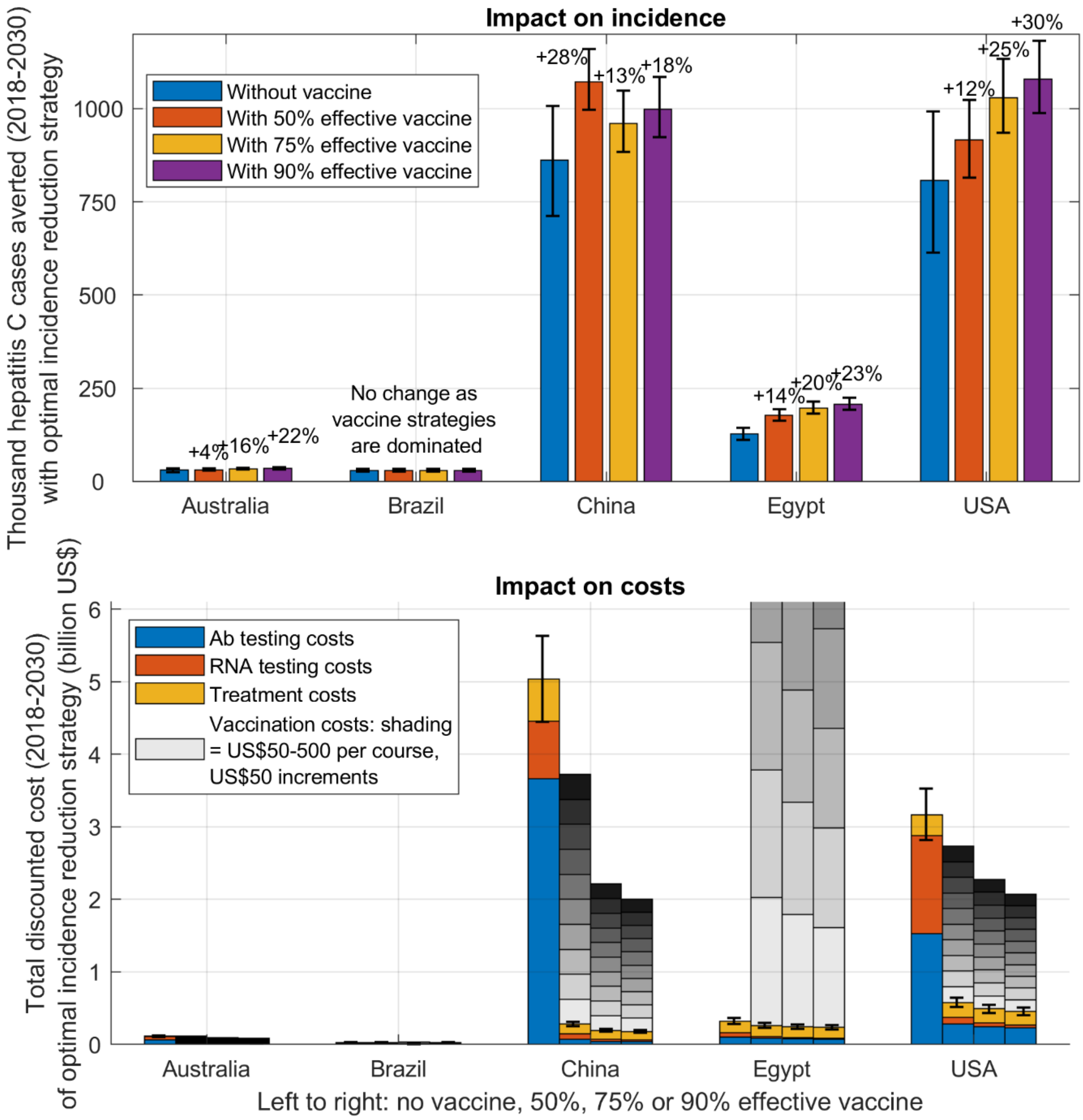

Figure S10: Potential impact of a hepatitis C vaccine in Australia, Brazil, China, Egypt and the USA. Top panel: cumulative cases averted 2018-2030 using the optimal strategy without a vaccine (blue) and with a 50%, 75% or 90% efficacious vaccine (red, yellow and purple, respectively). Bottom panel: total discounted costs of the optimal incidence reduction strategy without a vaccine (left bars) and with a 50%, 75% or 90% efficacious vaccine (right bars). Lightest grey shading represents total vaccination costs at US$50 per vaccine course, with darker shadings representing total costs as the vaccine price per course increases in US$50 increments. Uncertainty bounds represent scenarios with 70% and 90% coverage of testing, treatment and vaccination compared to a base of 80%.

APPENDIX C: GENERAL SETTINGS

Settings considered

Independent models were run for settings with concentrated epidemics (25%, 50% or 75% hepatitis C prevalence among PWID), generalised epidemics (1%, 2%, 3%, 5%, 10%, 15%, 25% or 30% hepatitis C prevalence among the general community) and mixed epidemics (all combinations of hepatitis C prevalence among PWID and the general community). Parameters used for these settings are shown in Table S3.

Table S3: Parameters for the general settings considered

Characteristics of sample settings to be tested (all combinations of the below)		
Proportion of the population who inject drugs	0.24%	Global average from Global Hepatitis Report[7].
Additional injecting-related mortality	0.0235 per year	Mathers et al. systematic review[13]
Epidemic type	Concentrated/ generalised / mixed	In concentrated epidemic settings only transmission among PWID was modelled; in generalised epidemic settings only transmission among the general community was modelled; and in mixed epidemic settings transmission among PWID and the general community was modelled.
Prevalence among PWID	25/50/75%	
Prevalence in general population	1/2/3/5/10/15/20/ 25/30%	
Healthcare system coverage	70/80/90%	This parameter defines the coverage of testing / vaccination that could be achieved, and is used to derive uncertainty bounds for outcomes
Staffing cost per interaction (testing+/-vaccination and treatment)	US$11.34	Global average, based on two hours of provider time for interaction and any laboratory work. Average salary calculated as the population-weighted per capita gross domestic product (GDP)[8]. Assumes providers work 7 hours per day, 5 days per week and 45 weeks per year.

Results

Availability of a 75% efficacious vaccine had the potential to substantially improve the impact of incidence reduction strategies, with the greatest benefits in settings with higher initial prevalence (Figure S11).

If a 75% efficacious vaccine cost US$200 per course, the vaccine strategies dominated the non-vaccine strategies in medium and high prevalence concentrated epidemic settings (i.e. cost less and prevented more incident cases) and were included within the optimal test/treat strategies for PWID.

In these settings, the optimal incidence reduction strategies with a vaccine available required 32% and 52% fewer treatments than the optimal strategies without the vaccine, respectively (Table S5).

In low, medium and high prevalence concentrated epidemic settings, the vaccine strategies dominated the non-vaccine strategies provided a vaccine course cost under US$77, US$263 or US$236 per course, respectively (Figure S11).

In generalised epidemic settings, the optimal incidence reduction strategy without a vaccine was screening and treatment of the general community, and the optimal strategy with a vaccine was to deliver vaccination with the general community screening program as well as through an age-based program (Table S4). The incidence reduction target was not able to be reached in generalised epidemic settings (unless testing levels among the general community were unfeasibly high, such as having annual testing of the entire population rather than having the entire population screened by 2030). However, use of a vaccine was able to approximately double the achievable incidence reduction in these settings.

For generalised epidemic settings with initial prevalence in the general community under 3%, 10%, 20% or 30%, unless the vaccine was under US$2.00, US$2.91, US$4.31, US$5.86 per course, respectively, use of a vaccine resulted in higher total costs. This is because the large-scale vaccine delivery was not entirely offset by the reduced treatment requirements.

In mixed epidemic settings, the optimal strategies included targeted programs for PWID, as well as programs among the general community, as per the concentrated and generalised epidemic settings.

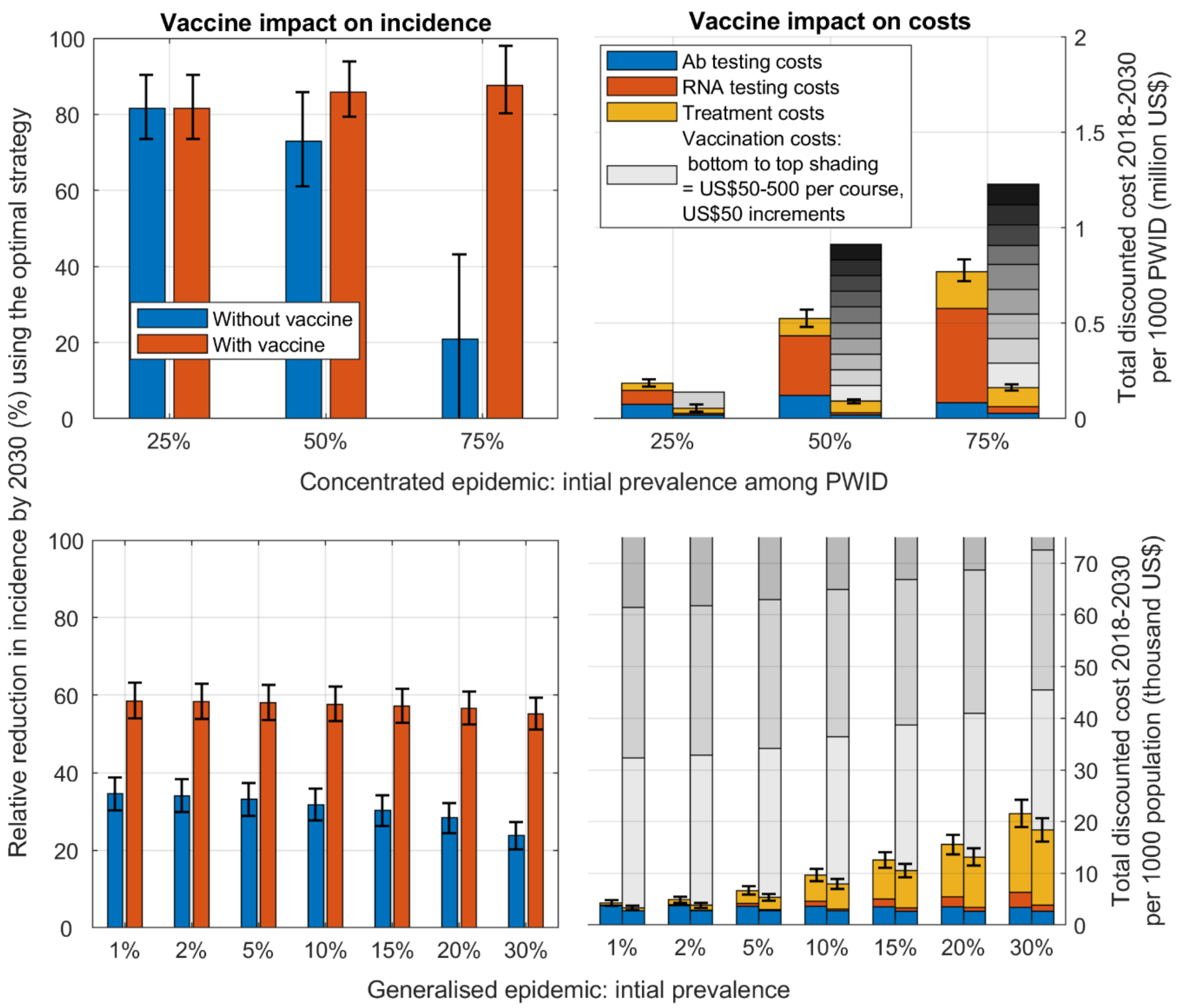

Figure S11: Potential impact of a 75% efficacious hepatitis C vaccine on incidence reduction strategies in concentrated and generalised epidemic settings. Left panels: incidence reduction in 2030 under the optimal incidence reduction strategies without a vaccine (blue bars) and with a 75% efficacious vaccine (red bars). Right panels: total discounted costs of the optimal incidence reduction strategies without a vaccine (left bars) and with a 75% efficacious vaccine available (right bars). Lightest grey shading represents total vaccination costs at US$50 per vaccine course, with darker shadings representing total costs as the vaccine price per course increases in US$50 increments. Uncertainty bounds represent scenarios with 70% and 90% coverage of testing, treatment and vaccination compared to a base of 80%.

Table S4: The optimal strategy to reduce incidence of hepatitis C by 2030 considering impact and cost per 1000 PWID for each of the settings.

Epidemic type	Prevalence among PWID	Prevalence among the general population	Optimal strategy: with a vaccine	Optimal strategy: no vaccine	Incidence reduction with vaccine^	Incidence reduction without vaccine^	Cost per 1000 target population* with US$200 vaccine (thousand US$)^	Cost per 1000 target population* without vaccine (thousand US$)^
Concentrated	25	N/A	Same as non-vaccine strategy	Annual PWID testing	82 (73, 90)	82 (73, 90)	$186 ($166, $204)	$186 ($166, $204)

Concentrated	50	N/A	2-yearly PWID testing + vaccination	6-monthly PWID testing	86 (78, 92)	73 (60, 85)	$420 ($368, $471)	$523 ($475, $566)
Concentrated	75	N/A	6-monthly PWID testing + vaccination	6-monthly PWID testing	88 (77, 95)	21 (-1, 49)	$677 ($595, $759)	$769 ($705, $818)
Generalised	N/A	1-10	General pop. testing +vaccination +Age vaccination	General pop. testing	58-59 (53, 63)	34-35 (29, 39)	$120-122 ($108, $134)	$4-10 ($4, $11)
Generalised	N/A	15	General pop. testing +vaccination +Age vaccination	General pop. testing	57 (53, 61)	30 (26, 34)	$123 ($111, $135)	$13 ($11, $14)
Generalised	N/A	20	General pop. testing +vaccination +Age vaccination	General pop. testing	57 (52, 61)	28 (25, 32)	$124 ($112, $136)	$16 ($14, $17)
Generalised	N/A	30	General pop. testing +vaccination +Age vaccination	General pop. testing	55 (51, 59)	24 (20, 27)	$127 ($114, $139)	$22 ($19, $24)
Mixed	25	1	6-monthly PWID testing + general pop. testing + vaccination + Age vac	6-monthly PWID testing + general pop. testing	73 (68, 77)	56 (50, 62)	$121 ($109, $133)	$5 ($5, $6)
Mixed	50	1	6-monthly PWID testing + general pop. testing + vaccination + Age vac	6-monthly PWID testing + general pop. testing	78 (73, 83)	60 (52, 69)	$121 ($109, $133)	$6 ($5, $6)
Mixed	75	1	6-monthly PWID testing + general pop. testing + vaccination + Age vac	6-monthly PWID testing + general pop. testing	79 (69, 86)	34 (18, 53)	$121 ($109, $133)	$6 ($6, $7)
Mixed	25	2-5	6-monthly PWID testing + general pop. testing + vaccination + Age vac	6-monthly PWID testing + general pop. testing	65-68 (60, 71)	45-50 (39, 55)	$121-123 ($110, $135)	$6-8 ($5, $9)
Mixed	50	2-5	6-monthly PWID testing + general pop. testing + vaccination + Age vac	6-monthly PWID testing + general pop. testing	67-72 (62, 77)	46-53 (40, 60)	$121-123 ($110, $135)	$6-9 ($6, $10)
Mixed	75	2-5	6-monthly PWID testing + general pop. testing + vaccination + Age vac	6-monthly PWID testing + general pop. testing	68-73 (61, 79)	39-40 (27, 52)	$122-123 ($110, $135)	$7-9 ($6, $10)
Mixed	25	10	6-monthly PWID testing + general pop. testing + vaccination + Age vac	6-monthly PWID testing + general pop. testing	64 (59, 68)	41 (36, 47)	$126 ($113, $138)	$12 ($10, $13)
Mixed	50	10	6-monthly PWID testing + general pop. testing + vaccination + Age vac	6-monthly PWID testing + general pop. testing	65 (60, 69)	42 (37, 48)	$126 ($113, $138)	$12 ($11, $14)
Mixed	75	10	6-monthly PWID testing + general pop. testing + vaccination + Age vac	6-monthly PWID testing + general pop. testing	65 (59, 70)	39 (33, 47)	$126 ($113, $138)	$13 ($11, $14)

*For concentrated epidemic settings, results are per 1000 PWID. For generalised and mixed epidemic settings, results are per 1000 population. Mixed epidemic settings assume 0.24% of the population injects drugs (see Table S3).

^Uncertainty bounds represent scenarios with 70% and 90% population coverage of testing, treatment and vaccination compared to a base of 80%.

Table S5: Total treatments, Ab tests, RNA tests and vaccinations required in each setting. Confidence intervals represent scenarios with 70% and 90% population coverage of testing, treatment and vaccination compared to a base of 80%. For concentrated epidemic settings, results are per 1000 PWID. For generalised and mixed epidemic settings, results are per 1000 population. Mixed epidemic settings assume 0.24% of the population injects drugs (see Table S3).

Epidemic type	Prevalence among PWID	Prevalence among the general population	Optimal strategy: with a vaccination	Optimal strategy: no vaccination	Total treatments with vaccine	Total treatments without vaccine	Total Ab tests with vaccine	Total Ab tests without vaccine	Total RNA tests with vaccine	Total RNA tests without vaccine	Total vaccinations
Concentrated	25	N/A	Same as non-vaccine strategy	Annual PWID testing	269 (252, 281)	269 (252, 281)	7,741 (6,722, 8,775)	7,741 (6,722, 8,775)	1,141 (1,035, 1,236)	1,141 (1,035, 1,236)	0
Concentrated	50	N/A	2-yearly PWID testing + vaccination	6-monthly PWID testing	425 (385, 463)	627 (611, 629)	1,900 (1,658, 2,143)	12,462 (10,606, 14,410)	202 (180, 224)	4,878 (4,474, 5,215)	2,054 (1,793, 2,316)
Concentrated	75	N/A	6-monthly PWID testing + vaccination	6-monthly PWID testing	677 (619, 730)	1,416 (1,413, 1,350)	2,683 (2,333, 3,036)	8,563 (7,018, 10,371)	529 (473, 581)	7,734 (7,016, 8,289)	3,150 (2,748, 3,552)
Generalised	N/A	1	general pop. testing + vaccination +Age vac	general pop. testing	4 (3, 4)	4 (3, 4)	278 (243, 313)	378 (331, 425)	1 (1, 1)	2 (1, 2)	728 (658, 799)
Generalised	N/A	2	general pop. testing + vaccination +Age vac	general pop. testing	8 (7, 8)	8 (7, 9)	277 (242, 312)	377 (330, 424)	1 (1, 1)	3 (3, 4)	727 (657, 797)
Generalised	N/A	3	general pop. testing + vaccination +Age vac	general pop. testing	11 (10, 13)	12 (10, 13)	277 (242, 312)	376 (329, 423)	2 (2, 2)	5 (4, 6)	725 (655, 795)
Generalised	N/A	5	general pop. testing + vaccination +Age vac	general pop. testing	19 (16, 21)	19 (17, 22)	276 (241, 311)	373 (327, 420)	3 (3, 4)	8 (7, 9)	721 (652, 791)
Generalised	N/A	10	general pop. testing + vaccination +Age vac	general pop. testing	38 (33, 42)	39 (34, 44)	273 (239, 308)	367 (321, 413)	6 (6, 7)	17 (14, 19)	713 (645, 782)
Generalised	N/A	15	general pop. testing + vaccination +Age vac	general pop. testing	56 (49, 63)	58 (51, 66)	271 (237, 305)	361 (316, 406)	10 (9, 11)	25 (22, 28)	705 (638, 772)
Generalised	N/A	20	general pop. testing + vaccination +Age vac	general pop. testing	75 (66, 84)	78 (69, 88)	268 (235, 303)	355 (311, 400)	13 (11, 15)	33 (29, 37)	697 (630, 763)
Generalised	N/A	30	general pop. testing + vaccination +Age vac	general pop. testing	113 (99, 127)	118 (103, 132)	264 (230, 297)	343 (300, 386)	19 (17, 22)	49 (43, 56)	680 (616, 744)
Mixed	25	1	6-monthly PWID testing + general pop. testing + vaccination + Age vac	6-monthly PWID testing + general pop. testing	5 (4, 6)	5 (5, 6)	284 (248, 320)	414 (362, 466)	1 (1, 1)	8 (7, 9)	734 (663, 805)
Mixed	50	1	6-monthly PWID testing + general pop. testing + vaccination + Age vac	6-monthly PWID testing + general pop. testing	5 (5, 6)	6 (5, 7)	284 (248, 320)	407 (355, 459)	2 (1, 2)	14 (13, 15)	734 (663, 805)

Mixed	75	1	6-monthly PWID testing + general pop. testing + vaccination + age vac	6-monthly PWID testing + general pop. testing	6<br>(5, 7)	8<br>(7, 8)	283<br>(247, 319)	398<br>(347, 449)	2<br>(2, 2)	21<br>(19, 22)	734<br>(663, 805)
Mixed	25	2	6-monthly PWID testing + general pop. testing + vaccination + age vac	6-monthly PWID testing + general pop. testing	10<br>(8, 11)	10<br>(9, 11)	284<br>(248, 320)	413<br>(361, 465)	2<br>(2, 2)	10<br>(9, 11)	733<br>(662, 805)
Mixed	50	2	6-monthly PWID testing + general pop. testing + vaccination + age vac	6-monthly PWID testing + general pop. testing	10<br>(9, 11)	11<br>(10, 12)	283<br>(247, 319)	406<br>(354, 458)	2<br>(2, 3)	16<br>(14, 17)	733<br>(662, 804)
Mixed	75	2	6-monthly PWID testing + general pop. testing + vaccination + age vac	6-monthly PWID testing + general pop. testing	11<br>(9, 12)	13<br>(11, 14)	283<br>(247, 319)	396<br>(346, 448)	3<br>(3, 3)	23<br>(21, 25)	733<br>(662, 804)
Mixed	25	3	6-monthly PWID testing + general pop. testing + vaccination + age vac	6-monthly PWID testing + general pop. testing	14<br>(13, 16)	15<br>(13, 17)	283<br>(247, 319)	412<br>(360, 464)	3<br>(2, 3)	12<br>(11, 13)	733<br>(662, 804)
Mixed	50	3	6-monthly PWID testing + general pop. testing + vaccination + age vac	6-monthly PWID testing + general pop. testing	15<br>(13, 16)	16<br>(14, 17)	283<br>(247, 319)	405<br>(353, 456)	3<br>(3, 4)	18<br>(16, 20)	733<br>(662, 804)
Mixed	75	3	6-monthly PWID testing + general pop. testing + vaccination + age vac	6-monthly PWID testing + general pop. testing	15<br>(13, 17)	17<br>(16, 19)	282<br>(246, 318)	395<br>(345, 446)	4<br>(3, 4)	25<br>(22, 27)	732<br>(662, 804)
Mixed	25	5	6-monthly PWID testing + general pop. testing + vaccination + age vac	6-monthly PWID testing + general pop. testing	24<br>(21, 26)	24<br>(21, 27)	282<br>(247, 318)	409<br>(358, 461)	4<br>(4, 5)	16<br>(14, 18)	731<br>(661, 802)
Mixed	50	5	6-monthly PWID testing + general pop. testing + vaccination + age vac	6-monthly PWID testing + general pop. testing	24<br>(21, 27)	25<br>(22, 28)	282<br>(246, 318)	402<br>(351, 453)	5<br>(4, 5)	22<br>(20, 24)	731<br>(660, 802)
Mixed	75	5	6-monthly PWID testing + general pop. testing + vaccination + age vac	6-monthly PWID testing + general pop. testing	24<br>(21, 27)	27<br>(24, 30)	281<br>(246, 317)	393<br>(343, 444)	5<br>(5, 6)	29<br>(26, 32)	731<br>(660, 802)
Mixed	25	10	6-monthly PWID testing + general pop. testing + vaccination + age vac	6-monthly PWID testing + general pop. testing	47<br>(41, 52)	48<br>(43, 54)	280<br>(244, 315)	403<br>(353, 454)	8<br>(7, 9)	26<br>(23, 29)	728<br>(658, 799)
Mixed	50	10	6-monthly PWID testing + general pop. testing + vaccination + age vac	6-monthly PWID testing + general pop. testing	47<br>(41, 53)	49<br>(43, 55)	279<br>(244, 315)	396<br>(346, 447)	9<br>(8, 10)	32<br>(29, 35)	728<br>(658, 798)
Mixed	75	10	6-monthly PWID testing + general pop. testing + vaccination + age vac	6-monthly PWID testing + general pop. testing	48<br>(42, 53)	51<br>(45, 56)	279<br>(243, 314)	387<br>(337, 437)	9<br>(8, 10)	39<br>(35, 43)	728<br>(657, 798)

APPENDIX D: SENSITIVITY ANALYSIS

When the vaccine efficacy was halved (37.5%) for individuals following successful treatment, the price per course had to be 2-20% cheaper in order to reduce the cost of elimination, but the optimal strategies were unchanged. This assumption had a much smaller impact on outcomes than the overall efficacy of the vaccine because the majority of vaccinations were delivered to uninfected people. For example, if the vaccine were 90% efficacious rather than 75%, the price point for the vaccine to reduce the costs of elimination was relaxed by 12-45%, whereas if the vaccine were only 50% efficacious, the price per course had to be 20-45% cheaper in order to reduce the cost of elimination (Table S6).

If no staff costs were included, meaning that the human resources associated with testing and treating to achieve eliminating were accounted for elsewhere, then the cost of a vaccine would have to be 20-46% cheaper in concentrated epidemic settings or 39-85% cheaper in generalised epidemic settings to reduce overall costs.

If the vaccine duration of protection was 5 years or 100 years (i.e. lifetime) compared to 10 years, then the price point was lowered by 13-26% (meaning the vaccine needed to be cheaper to save costs) or increased by 20-41% across the different prevalence settings, respectively.

Harm reduction scale-up only altered the optimal strategy in settings with low (<25%) prevalence among PWID: with a 60% scale-up of harm reduction, two-yearly testing rather than annual testing of PWID was sufficient to achieve the incidence reduction target in these settings. This meant that a vaccine did not reduce costs by as much and so had to be cheaper per course to reduce the cost of elimination. Scaling up harm reduction by 20%, 40% or 60% in non-vaccine scenarios meant that in settings with approximately 50% prevalence among PWID, the achievable incidence reduction increased from 73% to 75%, 76%, and 77% respectively, and in settings with approximately 75% prevalence among PWID the achievable incidence reduction increased from 21% to 26%, 30% and 35%.

Variations in the time between antibody test and RNA test, retention in care following a positive antibody test, and the time between RNA tests and treatment commencement had minimal impact of outcomes (Table S6).

Table S6: The price point at which a vaccine would reduce the cost of elimination under a variety of alternate assumptions. Vaccine is modelled to be 75% efficacious with a 10-year duration of protection, and be delivered with hepatitis C testing and following successful treatment. Results from mixed settings are not shown as they are similar to the results of concentrated + generalised.

Scenario	Concentrated epidemic settings			Generalised epidemic settings		
	25% prevalence among PWID	*50% prevalence among PWID*	*75% prevalence among PWID*	*1% prevalence among the general community*	*5% prevalence among the general community*	*10% prevalence among the general community*
Baseline	$77	$263	$236	$1.75	$3.59	$4.31

20% scale up of harm reduction among PWID	$76 (-1%)	$259 (-1%)	$283 (20%)	$1.75 (0%)	$3.59 (0%)	$4.31 (0%)
40% scale up of harm reduction among PWID	$75 (-3%)	$260 (-1%)	$279 (18%)	$1.75 (0%)	$3.59 (0%)	$4.31 (0%)
60% scale up of harm reduction among PWID	$27 (-65%)	$266 (1%)	$281 (19%)	$1.75 (0%)	$3.59 (0%)	$4.31 (0%)
90% efficacious vaccine rather than 75%	$88 (14%)	$294 (12%)	$335 (42%)	$2.54 (45%)	$5.2 (45%)	$6.25 (45%)
50% efficacious vaccine rather than 75%	$59 (-23%)	$210 (-20%)	$160 (-32%)	$0.96 (-45%)	$2.01 (-44%)	$2.42 (-44%)
Vaccine 50% less efficacious following successful treatment	$73 (-5%)	$244 (-7%)	$209 (-11%)	$1.71 (-2%)	$2.96 (-18%)	$3.44 (-20%)
No staff costs	$42 (-46%)	$180 (-32%)	$189 (-20%)	$0.26 (-85%)	$1.95 (-46%)	$2.62 (-39%)
Double staff costs	$113 (46%)	$346 (32%)	$283 (20%)	$3.25 (86%)	$5.22 (45%)	$5.99 (39%)
5 years duration of protection	$66 (-15%)	$229 (-13%)	$183 (-22%)	$1.3 (-26%)	$2.7 (-25%)	$3.25 (-25%)
100 years duration of protection	$95 (23%)	$315 (20%)	$319 (35%)	$2.47 (41%)	$4.98 (39%)	$5.96 (38%)
90 days between Ab and RNA tests rather than 60 days	$77 (0%)	$263 (0%)	$236 (0%)	$1.75 (0%)	$3.59 (0%)	$4.31 (0%)
Ab test and RNA test occurring on the same day (+100% follow-up)	$77 (0%)	$259 (-1%)	$569 (142%)	$1.78 (2%)	$4.04 (13%)	$4.89 (13%)
Average 60 days from diagnosis to treatment commencement rather than 30 days	$77 (0%)	$262 (0%)	$235 (0%)	$1.75 (0%)	$3.59 (0%)	$4.31 (0%)
70% follow-up RNA testing rather than 80%	$77 (0%)	$264 (1%)	$235 (0%)	$1.74 (-1%)	$3.38 (-6%)	$4.03 (-6%)
Half the testing positivity rate for the general community (testing at random)	$77 (0%)	$262 (0%)	$235 (0%)	$3.38 (93%)	$5.1 (42%)	$5.77 (34%)

APPENDIX E: COUNTRY SPECIFIC RESULTS

Table S7: Summary of country specific model estimates. Full results in Table S8 below.

Outcome	Without vaccine		With vaccine	
Optimal testing strategy among PWID (number of countries and percentage)	Number of countries	Percentage	Number of countries	Percentage
2-year	0	0%	47	28%
1-year	4	2%	26	16%
6-month	144	86%	94	56%
Countries where a vaccine reduced the testing frequency among PWID	N/A	N/A	69	41%
Countries where 80% reduction is achievable	8 (0-48)	5% (0-29%)	94 (15-113)	56% (9-68%)
Median incidence reduction by 2030	55%	(Range: 0-83%; IQR: 46-70%)	81%	(Range: 60-90%; IQR: 73-86%)
Price point for a vaccine to reduce the cost of elimination				
Concentrated epidemic	N/A	N/A	$247	(IQR: $204-442)
Mixed epidemic	N/A	N/A	$1.36	(IQR: $0.94-3.04)

Table S8: Country specific model estimates. Subregions: AFRO=Africa; EMRO=Eastern Mediterranean; EURO=Europe; PAHO=Americas; SEARO=South-East Asia; WPRO=Western Pacific.

Country	Cases averted with vaccine strategy compared to non-vaccine strategy	Optimal strategy: with a vaccine	Optimal strategy: no vaccine	Incidence reduction with vaccine	Incidence reduction without vaccine	Price point for a vaccine to save costs (US$0)	Cost with US$200 vaccine (million US$0)	Cost without vaccine (million US$0)	Difference in total cost (million US$0)
AFRO region, sorted by additional cases that could be averted if a vaccine were available									

Nigeria	53,156<br>(49,885, 54,858)	6-monthly PWID testing + GP testing + vaccination + age vaccination	6-monthly PWID testing + GP testing	68<br>(63, 73)	51<br>(44, 58)	$1.27	$10,797<br>($9,632, $11,964)	$248<br>($219, $277)	-$10,549<br>(-$11,686, -$9,413)
United Republic of Tanzania	24,895<br>(12,300, 34,942)	2-yearly PWID testing + vaccination	6-monthly PWID testing	87<br>(79, 93)	74<br>(61, 85)	$189.01	$139<br>($122, $156)	$133<br>($122, $143)	-$6<br>(-$14, $0)
Mozambique	19,032<br>(15,399, 21,057)	6-monthly PWID testing + GP testing + vaccination	6-monthly PWID testing + GP testing	84<br>(73, 91)	38<br>(19, 60)	$2.25	$1,465<br>($1,280, $1,651)	$34<br>($31, $37)	-$1,431<br>(-$1,614, -$1,250)
South Africa	18,552<br>(15,269, 20,814)	6-monthly PWID testing + GP testing + vaccination	6-monthly PWID testing + GP testing	83<br>(77, 87)	67<br>(56, 76)	$2.56	$3,663<br>($3,199, $4,129)	$138<br>($122, $153)	-$3,525<br>(-$3,976, -$3,078)
Ethiopia	15,119<br>(12,845, 16,654)	6-monthly PWID testing + GP testing + vaccination + age vaccination	6-monthly PWID testing + GP testing	79<br>(73, 83)	62<br>(53, 71)	$0.89	$6,011<br>($5,344, $6,679)	$78<br>($69, $86)	-$5,933<br>(-$6,593, -$5,275)
Ghana	10,513<br>(9,136, 11,301)	6-monthly PWID testing + GP testing + vaccination + age vaccination	6-monthly PWID testing + GP testing	71<br>(64, 77)	48<br>(38, 59)	$1.40	$1,736<br>($1,543, $1,930)	$39<br>($35, $44)	-$1,697<br>(-$1,887, -$1,508)
Algeria	9,275<br>(8,300, 9,883)	6-monthly PWID testing + GP testing + vaccination + age vaccination	6-monthly PWID testing + GP testing	72<br>(67, 77)	56<br>(48, 64)	$1.56	$2,826<br>($2,512, $3,141)	$77<br>($68, $87)	-$2,749<br>(-$3,054, -$2,444)
Democratic Republic of the Congo	9,172<br>(9,023, 9,172)	6-monthly PWID testing + GP testing + vaccination + age vaccination	6-monthly PWID testing + GP testing	63<br>(57, 68)	46<br>(40, 52)	$0.68	$4,226<br>($3,757, $4,697)	$69<br>($61, $78)	-$4,157<br>(-$4,619, -$3,696)
Uganda	7,961<br>(7,186, 8,439)	6-monthly PWID testing + GP testing + vaccination + age vaccination	6-monthly PWID testing + GP testing	72<br>(66, 77)	55<br>(48, 63)	$0.91	$2,227<br>($1,985, $2,470)	$32<br>($29, $36)	-$2,195<br>(-$2,434, -$1,956)
Angola	5,087<br>(4,541, 5,429)	6-monthly PWID testing + GP testing + vaccination + age vaccination	6-monthly PWID testing + GP testing	73<br>(67, 77)	56<br>(48, 64)	$1.44	$1,565<br>($1,392, $1,739)	$39<br>($34, $43)	-$1,527<br>(-$1,696, -$1,358)
Burkina Faso	4,206<br>(3,872, 4,396)	6-monthly PWID testing + GP testing + vaccination + age vaccination	6-monthly PWID testing + GP testing	70<br>(64, 75)	53<br>(46, 60)	$0.98	$1,038<br>($924, $1,153)	$18<br>($16, $20)	-$1,021<br>(-$1,134, -$908)
Cameroon	3,941<br>(3,440, 4,270)	6-monthly PWID testing + GP testing + vaccination + age vaccination	6-monthly PWID testing + GP testing	76<br>(70, 80)	59<br>(50, 67)	$1.03	$1,358<br>($1,209, $1,507)	$22<br>($19, $24)	-$1,336<br>(-$1,483, -$1,190)
Chad	3,776<br>(3,511, 3,926)	6-monthly PWID testing + GP testing + vaccination + age vaccination	6-monthly PWID testing + GP testing	70<br>(64, 75)	53<br>(45, 60)	$0.93	$802<br>($718, $886)	$12<br>($11, $14)	-$790<br>(-$873, -$707)
Zambia	3,672<br>(3,268, 3,923)	6-monthly PWID testing + GP testing + vaccination + age vaccination	6-monthly PWID testing + GP testing	73<br>(67, 77)	55<br>(47, 63)	$1.09	$939<br>($837, $1,042)	$16<br>($14, $18)	-$923<br>(-$1,023, -$823)
Niger	3,426<br>(3,062, 3,655)	6-monthly PWID testing + GP testing + vaccination + age vaccination	6-monthly PWID testing + GP testing	73<br>(67, 77)	56<br>(48, 64)	$0.88	$1,039<br>($924, $1,153)	$14<br>($13, $16)	-$1,024<br>(-$1,137, -$911)
Mali	3,129<br>(2,795, 3,338)	6-monthly PWID testing + GP testing + vaccination + age vaccination	6-monthly PWID testing + GP testing	73<br>(67, 77)	56<br>(48, 64)	$0.96	$951<br>($846, $1,057)	$15<br>($13, $16)	-$937<br>(-$1,040, -$833)
Senegal	2,930<br>(2,619, 3,125)	6-monthly PWID testing + GP testing + vaccination + age vaccination	6-monthly PWID testing + GP testing	73<br>(67, 77)	56<br>(48, 64)	$0.99	$887<br>($789, $985)	$14<br>($13, $16)	-$873<br>(-$970, -$777)
Kenya	2,887<br>(1,524, 3,961)	2-yearly PWID testing + vaccination	6-monthly PWID testing	85<br>(77, 92)	71<br>(58, 83)	$209.06	$13<br>($11, $15)	$13<br>($12, $14)	$0<br>($0, $1)
Zimbabwe	2,808<br>(2,727, 2,830)	6-monthly PWID testing + GP testing + vaccination + age vaccination	6-monthly PWID testing + GP testing	68<br>(62, 72)	52<br>(46, 58)	$0.76	$979<br>($873, $1,084)	$14<br>($13, $16)	-$964<br>(-$1,068, -$861)

Malawi	2,774 (2,701, 2,790)	6-monthly PWID testing + GP testing + vaccination + age vaccination	6-monthly PWID testing + GP testing	67 (62, 72)	52 (46, 57)	$0.61	$1,028 ($916, $1,139)	$12 ($11, $14)	-$1,015 (-$1,126, -$905)
Ivory Coast	2,580 (2,477, 2,650)	6-monthly PWID testing + GP testing + vaccination + age vaccination	6-monthly PWID testing + GP testing	61 (56, 66)	44 (39, 50)	$0.57	$1,362 ($1,210, $1,515)	$21 ($18, $23)	-$1,342 (-$1,492, -$1,192)
Guinea	2,440 (2,188, 2,597)	6-monthly PWID testing + GP testing + vaccination + age vaccination	6-monthly PWID testing + GP testing	72 (67, 77)	56 (48, 63)	$0.93	$721 ($642, $801)	$11 ($10, $12)	-$710 (-$789, -$632)
Burundi	2,395 (2,189, 2,518)	6-monthly PWID testing + GP testing + vaccination + age vaccination	6-monthly PWID testing + GP testing	71 (65, 76)	54 (47, 61)	$0.85	$602 ($538, $667)	$8 ($7, $9)	-$594 (-$658, -$530)
Gabon	2,321 (2,264, 2,321)	6-monthly PWID testing + GP testing + vaccination + age vaccination	6-monthly PWID testing + GP testing	62 (56, 67)	42 (35, 49)	$3.39	$131 ($116, $146)	$10 ($9, $12)	-$121 (-$134, -$107)
Benin	2,160 (1,941, 2,296)	6-monthly PWID testing + GP testing + vaccination + age vaccination	6-monthly PWID testing + GP testing	72 (67, 77)	56 (48, 63)	$0.95	$628 ($559, $697)	$10 ($9, $11)	-$618 (-$686, -$551)
Rwanda	1,815 (1,782, 1,815)	6-monthly PWID testing + GP testing + vaccination + age vaccination	6-monthly PWID testing + GP testing	64 (59, 69)	47 (41, 53)	$0.55	$720 ($641, $799)	$9 ($8, $10)	-$711 (-$789, -$633)
Liberia	1,162 (1,066, 1,220)	6-monthly PWID testing + GP testing + vaccination + age vaccination	6-monthly PWID testing + GP testing	71 (66, 76)	54 (47, 61)	$0.87	$279 ($250, $308)	$4 ($3, $4)	-$275 (-$304, -$246)
Mauritania	1,046 (954, 1,102)	6-monthly PWID testing + GP testing + vaccination + age vaccination	6-monthly PWID testing + GP testing	71 (66, 76)	55 (47, 62)	$1.00	$267 ($239, $296)	$4 ($4, $5)	-$263 (-$291, -$235)
Togo	1,043 (1,024, 1,043)	6-monthly PWID testing + GP testing + vaccination + age vaccination	6-monthly PWID testing + GP testing	66 (60, 71)	50 (44, 56)	$0.58	$447 ($398, $497)	$6 ($5, $6)	-$442 (-$491, -$393)
Sierra Leone	1,001 (958, 1,017)	6-monthly PWID testing + GP testing + vaccination + age vaccination	6-monthly PWID testing + GP testing	66 (60, 71)	49 (43, 56)	$0.58	$429 ($381, $477)	$5 ($5, $6)	-$423 (-$471, -$376)
Republic of the Congo	945 (841, 1,010)	6-monthly PWID testing + GP testing + vaccination + age vaccination	6-monthly PWID testing + GP testing	73 (67, 78)	56 (48, 64)	$2.62	$302 ($269, $336)	$14 ($13, $16)	-$288 (-$320, -$256)
Swaziland	799 (682, 904)	6-monthly PWID testing + GP testing + vaccination + age vaccination	GP testing	70 (58, 80)	13 (12, 15)	$0.21	$86 ($76, $95)	$2 ($1, $2)	-$84 (-$94, -$75)
Madagascar	631 (605, 650)	6-monthly PWID testing + GP testing + vaccination + age vaccination	6-monthly PWID testing + GP testing	62 (57, 67)	45 (40, 51)	$0.28	$1,481 ($1,318, $1,644)	$9 ($8, $10)	-$1,472 (-$1,634, -$1,310)
Namibia	567 (511, 601)	6-monthly PWID testing + GP testing + vaccination + age vaccination	6-monthly PWID testing + GP testing	72 (66, 77)	55 (48, 63)	$1.64	$161 ($143, $178)	$5 ($4, $5)	-$156 (-$173, -$139)
Botswana	497 (443, 531)	6-monthly PWID testing + GP testing + vaccination + age vaccination	6-monthly PWID testing + GP testing	73 (67, 78)	56 (48, 64)	$2.13	$157 ($139, $174)	$6 ($5, $7)	-$151 (-$168, -$134)
Mauritius	490 (242, 689)	2-yearly PWID testing + vaccination	6-monthly PWID testing	87 (79, 93)	74 (61, 85)	$271.02	$3 ($3, $3)	$4 ($3, $4)	$1 ($1, $1)
Lesotho	464 (414, 495)	6-monthly PWID testing + GP testing + vaccination + age vaccination	6-monthly PWID testing + GP testing	73 (67, 77)	56 (48, 64)	$1.01	$141 ($125, $157)	$2 ($2, $3)	-$139 (-$154, -$123)
Guinea-Bissau	426 (388, 449)	6-monthly PWID testing + GP testing + vaccination + age vaccination	6-monthly PWID testing + GP testing	71 (66, 76)	55 (47, 62)	$0.91	$110 ($98, $122)	$2 ($1, $2)	-$108 (-$120, -$97)
Gambia	368 (327, 395)	6-monthly PWID testing + GP testing + vaccination + age vaccination	6-monthly PWID testing + GP testing	74 (68, 78)	57 (49, 65)	$0.87	$114 ($102, $127)	$2 ($1, $2)	-$113 (-$125, -$101)

Equatorial Guinea	260<br>(232, 277)	6-monthly PWID testing + GP testing + vaccination + age vaccination	6-monthly PWID testing + GP testing	73<br>(67, 77)	56<br>(48, 64)	$2.47	$80<br>($71, $88)	$4<br>($3, $4)	-$76<br>(-$85, -$68)
Eritrea	171<br>(84, 240)	2-yearly PWID testing + vaccination	6-monthly PWID testing	87<br>(79, 93)	74<br>(61, 85)	$195.50	$1<br>($1, $1)	$1<br>($1, $1)	$0<br>($0, $0)
Central African Republic	167<br>(83, 235)	2-yearly PWID testing + vaccination	6-monthly PWID testing	87<br>(79, 93)	73<br>(61, 85)	$185.59	$1<br>($1, $1)	$1<br>($1, $1)	$0<br>($0, $0)
Comoros	159<br>(142, 170)	6-monthly PWID testing + GP testing + vaccination + age vaccination	6-monthly PWID testing + GP testing	73<br>(67, 77)	56<br>(48, 64)	$0.96	$48<br>($43, $54)	$1<br>($1, $1)	-$48<br>(-$53, -$42)
EMRO region, sorted by additional cases that could be averted if a vaccine were available									
Pakistan	435,128<br>(374,684, 488,308)	6-monthly PWID testing + GP testing + vaccination + age vaccination	GP testing	70<br>(59, 80)	14<br>(12, 15)	**	$12,786<br>($11,362, $14,214)	$351<br>($308, $395)	-$12,435<br>(-$13,818, -$11,054)
Egypt	69,543<br>(68,380, 69,543)	6-monthly PWID testing + GP testing + vaccination + age vaccination	6-monthly PWID testing + GP testing	65<br>(59, 70)	48<br>(42, 54)	$2.46	$6,434<br>($5,718, $7,152)	$324<br>($285, $362)	-$6,111<br>(-$6,790, -$5,433)
Sudan	26,343<br>(22,804, 28,493)	6-monthly PWID testing + GP testing + vaccination + age vaccination	6-monthly PWID testing + GP testing	73<br>(67, 78)	52<br>(42, 62)	$2.65	$2,373<br>($2,109, $2,636)	$90<br>($80, $100)	-$2,282<br>(-$2,536, -$2,029)
Morocco	21,096<br>(17,348, 23,145)	6-monthly PWID testing + GP testing + vaccination + age vaccination	6-monthly PWID testing + GP testing	79<br>(70, 86)	40<br>(23, 59)	$1.95	$2,511<br>($2,236, $2,787)	$65<br>($58, $72)	-$2,447<br>(-$2,715, -$2,179)
Somalia	13,584<br>(11,564, 14,642)	6-monthly PWID testing + GP testing + vaccination + age vaccination	6-monthly PWID testing + GP testing	73<br>(65, 79)	42<br>(29, 56)	$2.58	$789<br>($702, $875)	$26<br>($23, $28)	-$763<br>(-$847, -$679)
Syria	13,010<br>(11,390, 13,979)	6-monthly PWID testing + GP testing + vaccination + age vaccination	6-monthly PWID testing + GP testing	72<br>(65, 77)	51<br>(42, 61)	$3.46	$1,171<br>($1,039, $1,303)	$64<br>($56, $71)	-$1,107<br>(-$1,232, -$983)
South Sudan	12,361<br>(10,507, 13,335)	6-monthly PWID testing + GP testing + vaccination + age vaccination	6-monthly PWID testing + GP testing	73<br>(65, 79)	41<br>(29, 56)	$2.89	$727<br>($647, $807)	$27<br>($24, $30)	-$699<br>(-$777, -$622)
United Arab Emirates	12,138<br>(9,908, 13,358)	6-monthly PWID testing + GP testing + vaccination	6-monthly PWID testing + GP testing	80<br>(71, 87)	39<br>(21, 59)	$10.71	$870<br>($760, $980)	$146<br>($128, $164)	-$723<br>(-$816, -$631)
Saudi Arabia	6,381<br>(3,625, 8,511)	2-yearly PWID testing + vaccination	6-monthly PWID testing	84<br>(75, 91)	67<br>(52, 81)	$406.45	$24<br>($21, $27)	$41<br>($37, $45)	$18<br>($16, $18)
Kuwait	6,084<br>(5,210, 6,537)	6-monthly PWID testing + GP testing + vaccination + age vaccination	6-monthly PWID testing + GP testing	72<br>(65, 78)	42<br>(30, 55)	$8.60	$367<br>($327, $407)	$47<br>($42, $53)	-$320<br>(-$355, -$285)
Afghanistan	5,119<br>(1,855, 7,784)	2-yearly PWID testing + vaccination	6-monthly PWID testing	89<br>(82, 94)	79<br>(69, 88)	$145.74	$56<br>($49, $63)	$43<br>($39, $46)	-$13<br>(-$17, -$10)
Yemen	4,644<br>(4,079, 5,033)	6-monthly PWID testing + GP testing + vaccination + age vaccination	6-monthly PWID testing + GP testing	79<br>(74, 83)	66<br>(59, 72)	$1.18	$1,685<br>($1,500, $1,871)	$28<br>($24, $31)	-$1,658<br>(-$1,840, -$1,475)
Qatar	4,429<br>(3,911, 4,856)	6-monthly PWID testing + GP testing + vaccination + age vaccination	GP testing	78<br>(67, 87)	8<br>(7, 9)	$7.88	$272<br>($241, $304)	$52<br>($46, $59)	-$220<br>(-$245, -$196)
Iraq	2,816<br>(1,310, 4,026)	2-yearly PWID testing + vaccination	6-monthly PWID testing	87<br>(80, 93)	75<br>(63, 86)	$213.85	$19<br>($17, $21)	$20<br>($18, $22)	$1<br>($0, $2)
Iran	2,609<br>(2,480, 2,714)	6-monthly PWID testing + GP testing + vaccination + age vaccination	6-monthly PWID testing + GP testing	61<br>(55, 66)	44<br>(38, 49)	$1.48	$6,213<br>($5,531, $6,897)	$157<br>($137, $176)	-$6,056<br>(-$6,721, -$5,393)

Tunisia	2,228 (2,043, 2,345)	6-monthly PWID testing + GP testing + vaccination + age vaccination	6-monthly PWID testing + GP testing	74 (69, 78)	61 (54, 67)	$1.66	$844 ($751, $937)	$22 ($20, $25)	-$821 (-$912, -$731)
Bahrain	1,668 (1,356, 1,840)	6-monthly PWID testing + GP testing + vaccination	6-monthly PWID testing + GP testing	82 (72, 89)	38 (20, 59)	$7.42	$116 ($102, $131)	$13 ($12, $15)	-$103 (-$116, -$90)
Djibouti	1,056 (890, 1,144)	6-monthly PWID testing + GP testing + vaccination + age vaccination	6-monthly PWID testing + GP testing	74 (66, 80)	41 (28, 56)	$3.80	$66 ($58, $73)	$4 ($3, $4)	-$62 (-$69, -$55)
Cyprus	468 (450, 480)	6-monthly PWID testing + GP testing + vaccination + age vaccination	6-monthly PWID testing + GP testing	62 (56, 67)	45 (39, 50)	$5.08	$93 ($83, $104)	$10 ($8, $11)	-$84 (-$93, -$74)
Lebanon	410 (169, 605)	2-yearly PWID testing + vaccination	6-monthly PWID testing	88 (81, 94)	77 (67, 87)	$232.24	$4 ($3, $4)	$4 ($4, $5)	$0 ($0, $1)
Oman	352 (322, 371)	6-monthly PWID testing + GP testing + vaccination + age vaccination	6-monthly PWID testing + GP testing	76 (71, 80)	64 (57, 69)	$3.87	$369 ($327, $410)	$24 ($21, $27)	-$345 (-$383, -$306)
Jordan		Same as non-vaccine strategy	Annual PWID testing		83 (75, 90)	$46.55		$1 ($1, $2)	
EURO region, sorted by additional cases that could be averted if a vaccine were available									
Italy	166,062 (128,922, 188,377)	Annual PWID testing + vaccination	6-monthly PWID testing	84 (74, 93)	36 (15, 61)	$435.83	$203 ($179, $227)	$360 ($323, $393)	$156 ($144, $165)
Ukraine	119,209 (90,994, 137,628)	Annual PWID testing + vaccination	6-monthly PWID testing	87 (78, 94)	50 (31, 70)	$213.35	$159 ($140, $178)	$168 ($154, $178)	$8 ($0, $14)
Uzbekistan	99,338 (87,887, 108,847)	6-monthly PWID testing + GP testing + vaccination + age vaccination	GP testing	74 (64, 82)	13 (11, 14)	**	$2,371 ($2,106, $2,637)	$76 ($67, $86)	-$2,295 (-$2,551, -$2,040)
Georgia	36,868 (27,990, 42,811)	Annual PWID testing + vaccination	6-monthly PWID testing	88 (79, 94)	54 (36, 73)	$216.59	$55 ($49, $62)	$59 ($54, $63)	$4 ($1, $5)
Turkey	34,206 (18,597, 46,425)	2-yearly PWID testing + vaccination	6-monthly PWID testing	85 (77, 92)	69 (56, 83)	$307.15	$147 ($129, $165)	$208 ($188, $226)	$60 ($58, $60)
United Kingdom	23,422 (12,642, 31,876)	2-yearly PWID testing + vaccination	6-monthly PWID testing	85 (77, 92)	70 (56, 83)	$588.05	$123 ($108, $138)	$277 ($247, $307)	$154 ($139, $169)
Kyrgyzstan	21,044 (16,965, 23,276)	6-monthly PWID testing + vaccination	6-monthly PWID testing	88 (78, 95)	27 (4, 54)	$200.39	$18 ($16, $20)	$18 ($17, $19)	$0 (-$1, $1)
Poland	17,962 (9,879, 24,275)	2-yearly PWID testing + vaccination	6-monthly PWID testing	85 (76, 92)	69 (55, 82)	$324.32	$74 ($65, $83)	$108 ($98, $118)	$35 ($33, $35)
Kazakhstan	16,004 (9,439, 21,001)	2-yearly PWID testing + vaccination	6-monthly PWID testing	83 (73, 90)	64 (48, 79)	$303.07	$47 ($41, $53)	$66 ($60, $71)	$18 ($18, $18)
Romania	14,452 (12,198, 16,002)	6-monthly PWID testing + GP testing + vaccination	6-monthly PWID testing + GP testing	80 (75, 85)	67 (58, 75)	$6.30	$1,371 ($1,198, $1,545)	$109 ($96, $121)	-$1,262 (-$1,424, -$1,102)
Israel	13,577 (10,368, 15,664)	Annual PWID testing + vaccination	6-monthly PWID testing	87 (77, 94)	48 (29, 69)	$461.00	$22 ($19, $25)	$41 ($36, $45)	$19 ($17, $20)
Denmark	13,545 (11,543, 15,321)	6-monthly PWID testing + vaccination	**	79 (65, 91)	0 (0, 0)	**	$14 ($13, $16)	$0 ($0, $0)	-$14 (-$16, -$13)

France	12,678 (7,583, 16,530)	2-yearly PWID testing + vaccination	6-monthly PWID testing	82 (73, 90)	62 (46, 78)	$588.89	$42 ($37, $47)	$93 ($83, $103)	$52 ($47, $56)
Portugal	11,864 (10,254, 13,256)	6-monthly PWID testing + vaccination	**	81 (68, 92)	0 (0, 0)	**	$11 ($9, $12)	$0 ($0, $0)	-$11 (-$12, -$9)
Latvia	9,421 (8,014, 10,676)	6-monthly PWID testing + vaccination	**	79 (65, 91)	0 (0, 0)	**	$8 ($7, $9)	$0 ($0, $0)	-$8 (-$9, -$7)
Bulgaria	7,639 (5,858, 8,775)	Annual PWID testing + vaccination	6-monthly PWID testing	86 (76, 93)	46 (26, 67)	$258.44	$10 ($9, $11)	$12 ($11, $13)	$2 ($2, $2)
Switzerland	6,891 (5,345, 7,824)	Annual PWID testing + vaccination	6-monthly PWID testing	84 (74, 93)	37 (15, 61)	$775.51	$11 ($10, $12)	$27 ($24, $30)	$16 ($14, $18)
Estonia	6,491 (5,526, 7,348)	6-monthly PWID testing + vaccination	**	79 (65, 91)	0 (0, 0)	**	$6 ($5, $6)	$0 ($0, $0)	-$6 (-$6, -$5)
Sweden	6,421 (5,206, 7,060)	6-monthly PWID testing + vaccination	6-monthly PWID testing	87 (77, 95)	23 (0, 51)	$590.69	$7 ($6, $8)	$12 ($11, $14)	$6 ($5, $6)
Belarus	5,347 (3,133, 7,037)	2-yearly PWID testing + vaccination	6-monthly PWID testing	83 (74, 90)	64 (49, 79)	$274.22	$16 ($14, $18)	$21 ($19, $22)	$5 ($4, $5)
Germany	5,276 (2,105, 7,854)	2-yearly PWID testing + vaccination	6-monthly PWID testing	88 (82, 94)	78 (67, 88)	$551.32	$63 ($55, $71)	$137 ($122, $153)	$74 ($66, $82)
Finland	5,263 (3,983, 6,131)	Annual PWID testing + vaccination	6-monthly PWID testing	88 (80, 94)	56 (38, 74)	$487.12	$11 ($9, $12)	$20 ($18, $22)	$10 ($9, $10)
Azerbaijan	5,164 (2,948, 6,871)	2-yearly PWID testing + vaccination	6-monthly PWID testing + GP testing	83 (74, 91)	66 (51, 80)	$584.78	$17 ($15, $19)	$43 ($39, $47)	$26 ($23, $28)
Norway	4,350 (3,378, 4,934)	Annual PWID testing + vaccination	6-monthly PWID testing	85 (74, 93)	38 (17, 62)	$699.03	$6 ($6, $7)	$15 ($13, $17)	$9 ($8, $9)
Tajikistan	3,832 (2,842, 4,579)	6-monthly PWID testing + vaccination	6-monthly PWID testing + GP testing	80 (76, 83)	72 (61, 81)	$239.76	$14 ($12, $16)	$17 ($15, $18)	$2 ($2, $2)
Macedonia	3,725 (2,840, 4,304)	Annual PWID testing + vaccination	6-monthly PWID testing	87 (78, 94)	50 (31, 70)	$237.68	$5 ($5, $6)	$6 ($5, $6)	$1 ($1, $1)
Belgium	3,566 (2,092, 4,690)	2-yearly PWID testing + vaccination	6-monthly PWID testing	83 (74, 90)	64 (49, 79)	$621.88	$13 ($11, $15)	$30 ($27, $33)	$17 ($16, $19)
Czechia	3,257 (1,415, 4,745)	2-yearly PWID testing + vaccination	6-monthly PWID testing	88 (81, 94)	77 (65, 87)	$336.63	$29 ($25, $32)	$44 ($39, $48)	$15 ($14, $16)
Austria	3,030 (1,835, 3,927)	2-yearly PWID testing + vaccination	6-monthly PWID testing	81 (72, 90)	61 (44, 77)	$670.22	$10 ($9, $11)	$24 ($21, $26)	$14 ($13, $15)
Libya	2,454 (2,120, 2,751)	6-monthly PWID testing + GP testing + vaccination + age vaccination	GP testing	69 (59, 78)	18 (15, 20)	$3.96	$488 ($435, $542)	$43 ($38, $49)	-$445 (-$493, -$398)
Turkmenistan	2,163 (1,149, 2,962)	2-yearly PWID testing + vaccination	6-monthly PWID testing	85 (77, 92)	71 (57, 83)	$258.31	$10 ($9, $11)	$12 ($11, $13)	$2 ($2, $2)
Moldova	1,896 (1,541, 2,147)	6-monthly PWID testing + GP testing + vaccination	6-monthly PWID testing + GP testing	87 (81, 91)	71 (61, 80)	$2.92	$261 ($228, $295)	$9 ($8, $10)	-$253 (-$285, -$221)

Luxembourg	1,868 (1,632, 2,068)	6-monthly PWID testing + vaccination	**	83 (71, 93)	0 (0, 0)	**	$2 ($2, $3)	$0 ($0, $0)	-$2 (-$3, -$2)
Lithuania	1,699 (1,375, 1,906)	6-monthly PWID testing + GP testing + vaccination	6-monthly PWID testing + GP testing	83 (75, 88)	57 (42, 71)	$5.23	$196 ($171, $221)	$16 ($14, $18)	-$180 (-$203, -$157)
Spain	1,149 (655, 1,532)	2-yearly PWID testing + vaccination	6-monthly PWID testing	84 (75, 91)	67 (52, 81)	$467.89	$4 ($4, $5)	$8 ($7, $9)	$4 ($4, $4)
Ireland	1,122 (654, 1,481)	2-yearly PWID testing + vaccination	6-monthly PWID testing	83 (74, 91)	65 (49, 80)	$837.35	$5 ($4, $5)	$13 ($12, $15)	$8 ($8, $9)
Hungary	1,108 (1,006, 1,171)	6-monthly PWID testing + GP testing + vaccination + age vaccination	6-monthly PWID testing + GP testing	72 (67, 77)	57 (50, 64)	$2.78	$728 ($648, $809)	$39 ($34, $44)	-$689 (-$765, -$613)
Bosnia and Herzegovina	649 (256, 968)	2-yearly PWID testing + vaccination	6-monthly PWID testing	89 (82, 94)	78 (67, 88)	$194.23	$6 ($6, $7)	$6 ($6, $7)	$0 ($0, $0)
Armenia	526 (211, 782)	2-yearly PWID testing + vaccination	6-monthly PWID testing + GP testing	88 (81, 93)	77 (67, 87)	$448.17	$5 ($4, $5)	$10 ($9, $11)	$5 ($5, $6)
Croatia	493 (253, 683)	2-yearly PWID testing + vaccination	6-monthly PWID testing	86 (78, 92)	72 (59, 84)	$305.32	$3 ($2, $3)	$4 ($3, $4)	$1 ($1, $1)
Slovakia	432 (105, 704)	2-yearly PWID testing + vaccination	6-monthly PWID testing	90 (84, 95)	81 (72, 90)	$273.03	$8 ($7, $9)	$10 ($9, $11)	$2 ($2, $2)
Greece	405 (209, 561)	2-yearly PWID testing + vaccination	6-monthly PWID testing	86 (78, 92)	72 (59, 84)	$358.97	$2 ($2, $2)	$3 ($3, $4)	$1 ($1, $1)
Netherlands	252 (127, 350)	2-yearly PWID testing + vaccination	6-monthly PWID testing	86 (78, 93)	73 (60, 85)	$620.48	$2 ($1, $2)	$4 ($3, $4)	$2 ($2, $2)
Iceland	51 (19, 77)	2-yearly PWID testing + vaccination	6-monthly PWID testing	89 (82, 94)	80 (68, 88)	$718.31	$1 ($1, $1)	$2 ($2, $2)	$1 ($1, $1)
Albania		Same as non-vaccine strategy	6-monthly PWID testing		81 (71, 89)	$163.79		$4 ($4, $5)	
Russia		Same as non-vaccine strategy	Annual PWID testing		80 (71, 89)	$81.58		$337 ($301, $371)	
Slovenia		Same as non-vaccine strategy	Annual PWID testing		82 (73, 90)	$127.3		$2 ($1, $2)	
PAHO region, sorted by additional cases that could be averted if a vaccine were available									
United States of America	220,603 (119,627, 299,808)	2-yearly PWID testing + vaccination	6-monthly PWID testing	85 (77, 92)	70 (56, 83)	$749.73	$1,206 ($1,059, $1,352)	$3,168 ($2,807, $3,517)	$1,962 ($1,748, $2,165)
Canada	149,364 (115,582, 170,033)	Annual PWID testing + vaccination	6-monthly PWID testing	85 (75, 93)	40 (19, 64)	$502.68	$202 ($178, $226)	$392 ($350, $430)	$189 ($172, $203)
Mexico	121,064 (102,928, 137,252)	6-monthly PWID testing + vaccination	**	79 (65, 91)	0 (0, 0)	**	$103 ($90, $115)	$0 ($0, $0)	-$103 (-$115, -$90)
Peru	70,210 (60,367, 78,801)	6-monthly PWID testing + vaccination	**	81 (67, 92)	0 (0, 0)	**	$58 ($51, $65)	$0 ($0, $0)	-$58 (-$65, -$51)

Chile	20,168 (15,457, 23,190)	Annual PWID testing + vaccination	6-monthly PWID testing	86 (77, 94)	47 (28, 69)	$294.42	$27 ($24, $30)	$36 ($33, $39)	$9 ($9, $9)
Guatemala	16,844 (12,876, 19,412)	Annual PWID testing + vaccination	6-monthly PWID testing	86 (77, 94)	48 (28, 69)	$233.48	$22 ($20, $25)	$25 ($23, $27)	$3 ($2, $3)
El Salvador	13,890 (12,261, 15,239)	6-monthly PWID testing + vaccination	**	85 (73, 94)	0 (0, 0)	**	$11 ($10, $12)	$0 ($0, $0)	-$11 (-$12, -$10)
Cuba	13,631 (10,421, 15,706)	Annual PWID testing + vaccination	6-monthly PWID testing	86 (77, 94)	48 (28, 69)	$273.61	$19 ($16, $21)	$24 ($22, $26)	$5 ($5, $5)
Dominican Republic	11,900 (9,108, 13,698)	Annual PWID testing + vaccination	6-monthly PWID testing	86 (77, 94)	48 (28, 69)	$248.61	$16 ($14, $17)	$18 ($17, $20)	$3 ($2, $3)
Haiti	11,741 (8,985, 13,519)	Annual PWID testing + vaccination	6-monthly PWID testing	86 (77, 94)	48 (28, 69)	$207.97	$15 ($13, $17)	$15 ($14, $16)	$0 ($0, $1)
Bolivia	11,589 (8,866, 13,347)	Annual PWID testing + vaccination	6-monthly PWID testing	86 (77, 94)	48 (28, 69)	$224.80	$15 ($13, $17)	$16 ($15, $18)	$1 ($1, $2)
Argentina	7,389 (3,926, 10,112)	2-yearly PWID testing + vaccination	6-monthly PWID testing + GP testing	85 (77, 92)	70 (57, 83)	$1,528.17	$34 ($30, $39)	$208 ($183, $232)	$173 ($153, $193)
Ecuador	3,397 (1,730, 4,720)	2-yearly PWID testing + vaccination	6-monthly PWID testing	86 (78, 93)	72 (60, 84)	$244.63	$18 ($16, $20)	$21 ($19, $23)	$3 ($3, $3)
Jamaica	3,363 (2,572, 3,873)	Annual PWID testing + vaccination	6-monthly PWID testing	86 (77, 94)	48 (28, 69)	$237.27	$4 ($4, $5)	$5 ($5, $5)	$1 ($0, $1)
Trinidad and Tobago	1,634 (1,250, 1,883)	Annual PWID testing + vaccination	6-monthly PWID testing	86 (77, 94)	48 (28, 69)	$314.63	$2 ($2, $3)	$3 ($3, $4)	$1 ($1, $1)
Panama	1,568 (923, 2,060)	2-yearly PWID testing + vaccination	6-monthly PWID testing	83 (73, 90)	64 (48, 79)	$358.17	$5 ($4, $5)	$8 ($7, $8)	$3 ($3, $3)
Honduras	1,557 (744, 2,208)	2-yearly PWID testing + vaccination	6-monthly PWID testing	87 (79, 93)	75 (62, 86)	$196.49	$10 ($8, $11)	$9 ($9, $10)	$0 (-$1, $0)
Venezuela	1,552 (292, 2,594)	2-yearly PWID testing + vaccination	6-monthly PWID testing	90 (84, 95)	82 (73, 90)	$204.44	$34 ($30, $38)	$35 ($31, $38)	$1 ($0, $1)
Paraguay	1,077 (1,009, 1,117)	6-monthly PWID testing + GP testing + vaccination + age vaccination	6-monthly PWID testing + GP testing	73 (68, 78)	60 (54, 66)	$1.87	$476 ($424, $527)	$13 ($12, $15)	-$462 (-$513, -$412)
Guyana	900 (689, 1,036)	Annual PWID testing + vaccination	6-monthly PWID testing	86 (77, 94)	48 (28, 69)	$232.64	$1 ($1, $1)	$1 ($1, $1)	$0 ($0, $0)
Nicaragua	757 (319, 1,111)	2-yearly PWID testing + vaccination	6-monthly PWID testing	88 (81, 94)	77 (66, 87)	$176.77	$7 ($6, $7)	$6 ($5, $6)	-$1 (-$1, $0)
Costa Rica	713 (691, 725)	6-monthly PWID testing + GP testing + vaccination + age vaccination	6-monthly PWID testing + GP testing	63 (58, 68)	47 (41, 52)	$3.69	$377 ($335, $418)	$21 ($19, $24)	-$355 (-$394, -$317)
Suriname	652 (499, 751)	Annual PWID testing + vaccination	6-monthly PWID testing	86 (77, 94)	48 (28, 69)	$242.88	$1 ($1, $1)	$1 ($1, $1)	$0 ($0, $0)
Uruguay	530 (457, 581)	6-monthly PWID testing + GP testing + vaccination	6-monthly PWID testing + GP testing	81 (76, 85)	70 (63, 76)	$4.73	$228 ($199, $257)	$18 ($15, $20)	-$210 (-$237, -$184)

Belize	401 (306, 462)	Annual PWID testing + vaccination	6-monthly PWID testing	86 (77, 94)	48 (28, 69)	$237.63	$1 ($0, $1)	$1 ($1, $1)	$0 ($0, $0)
Antigua and Barbuda	127 (97, 146)	Annual PWID testing + vaccination	6-monthly PWID testing	86 (77, 94)	47 (28, 69)	$298.76	$0 ($0, $0)	$0 ($0, $0)	$0 ($0, $0)
Brazil		Same as non-vaccine strategy	Annual PWID testing		80 (71, 89)	$80.9		$27 ($25, $30)	
Colombia		Same as non-vaccine strategy	6-monthly PWID testing		82 (73, 90)	$162.45		$45 ($41, $49)	
SEARO region, sorted by additional cases that could be averted if a vaccine were available									
Indonesia	161,966 (142,638, 177,769)	6-monthly PWID testing + GP testing + vaccination	GP testing	81 (69, 91)	3 (3, 4)	$0.31	$17,261 ($15,082, $19,449)	$344 ($301, $387)	-$16,917 (-$19,062, -$14,780)
India	126,103 (116,166, 131,797)	6-monthly PWID testing + GP testing + vaccination + age vaccination	6-monthly PWID testing + GP testing	70 (64, 75)	54 (47, 61)	$0.71	$92,119 ($81,830, $102,422)	$1,278 ($1,124, $1,432)	-$90,840 (-$100,991, -$80,707)
Thailand	48,553 (41,050, 55,270)	6-monthly PWID testing + GP testing + vaccination + age vaccination	GP testing	73 (59, 84)	7 (7, 8)	$0.69	$5,356 ($4,770, $5,943)	$146 ($128, $164)	-$5,210 (-$5,779, -$4,642)
Myanmar	21,698 (12,417, 28,859)	2-yearly PWID testing + vaccination	6-monthly PWID testing	83 (75, 91)	66 (51, 81)	$227.80	$68 ($60, $77)	$76 ($70, $81)	$8 ($4, $10)
Bangladesh	16,801 (15,374, 17,677)	6-monthly PWID testing + GP testing + vaccination + age vaccination	6-monthly PWID testing + GP testing	72 (67, 77)	58 (51, 65)	$0.72	$11,412 ($10,147, $12,678)	$149 ($131, $167)	-$11,263 (-$12,512, -$10,016)
Nepal	2,375 (2,261, 2,467)	6-monthly PWID testing + GP testing + vaccination + age vaccination	6-monthly PWID testing + GP testing	61 (55, 66)	43 (38, 49)	$0.42	$1,960 ($1,747, $2,174)	$18 ($16, $21)	-$1,942 (-$2,153, -$1,731)
Sri Lanka	1,387 (1,315, 1,444)	6-monthly PWID testing + GP testing + vaccination + age vaccination	6-monthly PWID testing + GP testing	60 (55, 66)	44 (38, 49)	$0.85	$1,483 ($1,317, $1,648)	$30 ($26, $34)	-$1,452 (-$1,615, -$1,291)
Bhutan	205 (170, 228)	6-monthly PWID testing + GP testing + vaccination + age vaccination	6-monthly PWID testing + GP testing	81 (74, 86)	60 (48, 71)	$1.38	$60 ($53, $66)	$1 ($1, $1)	-$58 (-$65, -$52)
Timor-Leste	10 (6, 13)	2-yearly PWID testing + vaccination	6-monthly PWID testing + GP testing	81 (72, 89)	62 (46, 78)	$5,776.47	$0 ($0, $0)	$1 ($1, $1)	$1 ($1, $1)
WPRO region, sorted by additional cases that could be averted if a vaccine were available									
Malaysia	237,068 (202,081, 268,075)	6-monthly PWID testing + vaccination	**	79 (65, 91)	0 (0, 0)	**	$197 ($173, $220)	$0 ($0, $0)	-$197 (-$220, -$173)
China	99,374 (26,976, 158,390)	2-yearly PWID testing + vaccination	6-monthly PWID testing + GP testing	82 (76, 88)	74 (64, 84)	$1,200.06	$1,001 ($878, $1,124)	$5,034 ($4,437, $5,622)	$4,033 ($3,559, $4,498)
South Korea	71,079 (61,905, 78,873)	6-monthly PWID testing + vaccination	**	83 (70, 93)	0 (0, 0)	**	$64 ($57, $72)	$0 ($0, $0)	-$64 (-$72, -$57)
Japan	26,772 (13,520, 37,302)	2-yearly PWID testing + vaccination	6-monthly PWID testing	86 (79, 93)	73 (60, 85)	$557.85	$176 ($155, $198)	$384 ($342, $426)	$208 ($187, $228)
Philippines	19,987 (17,425, 21,374)	6-monthly PWID testing + GP testing + vaccination + age vaccination	6-monthly PWID testing + GP testing	70 (63, 76)	46 (35, 57)	$1.06	$6,982 ($6,210, $7,755)	$137 ($121, $154)	-$6,844 (-$7,601, -$6,089)

Vietnam	18,745 (18,259, 18,964)	6-monthly PWID testing + GP testing + vaccination + age vaccination	6-monthly PWID testing + GP testing	64 (58, 69)	48 (42, 53)	$0.99	$7,154 ($6,366, $7,943)	$142 ($124, $159)	-$7,012 (-$7,784, -$6,242)
Papua New Guinea	8,832 (7,506, 10,017)	6-monthly PWID testing + vaccination	**	79 (64, 91)	0 (0, 0)	**	$7 ($6, $8)	$0 ($0, $0)	-$7 (-$8, -$6)
Cambodia	6,385 (6,024, 6,565)	6-monthly PWID testing + GP testing + vaccination + age vaccination	6-monthly PWID testing + GP testing	68 (63, 73)	51 (44, 58)	$1.18	$1,111 ($991, $1,230)	$24 ($21, $26)	-$1,087 (-$1,204, -$971)
Australia	3,747 (1,468, 5,602)	2-yearly PWID testing + vaccination	6-monthly PWID testing	89 (82, 94)	78 (67, 88)	$621.81	$48 ($42, $54)	$114 ($101, $127)	$66 ($58, $73)
Mongolia	2,938 (2,910, 2,938)	6-monthly PWID testing + GP testing + vaccination + age vaccination	6-monthly PWID testing + GP testing	64 (59, 69)	47 (41, 53)	$2.62	$223 ($199, $248)	$12 ($11, $14)	-$211 (-$234, -$188)
Laos	1,087 (968, 1,167)	6-monthly PWID testing + GP testing + vaccination + age vaccination	6-monthly PWID testing + GP testing	78 (73, 82)	65 (58, 71)	$1.37	$458 ($407, $508)	$9 ($8, $11)	-$448 (-$497, -$399)
Fiji	964 (776, 1,069)	6-monthly PWID testing + vaccination	6-monthly PWID testing	88 (78, 95)	29 (7, 56)	$265.96	$1 ($1, $1)	$1 ($1, $1)	$0 ($0, $0)
New Zealand	694 (239, 1,066)	2-yearly PWID testing + vaccination	6-monthly PWID testing	89 (83, 94)	79 (69, 88)	$512.43	$11 ($9, $12)	$22 ($19, $24)	$11 ($10, $13)
Singapore	653 (322, 918)	2-yearly PWID testing + vaccination	6-monthly PWID testing	87 (79, 93)	74 (61, 85)	$684.15	$5 ($4, $5)	$12 ($11, $14)	$7 ($7, $8)
Vanuatu	298 (253, 337)	6-monthly PWID testing + vaccination	**	79 (65, 91)	0 (0, 0)	**	$0 ($0, $0)	$0 ($0, $0)	$0 ($0, $0)
Solomon Islands	54 (27, 76)	2-yearly PWID testing + vaccination	6-monthly PWID testing	87 (79, 93)	73 (61, 85)	$200.79	$0 ($0, $0)	$0 ($0, $0)	$0 ($0, $0)
Brunei	49 (24, 68)	2-yearly PWID testing + vaccination	6-monthly PWID testing	87 (79, 93)	73 (61, 85)	$438.19	$0 ($0, $0)	$1 ($1, $1)	$0 ($0, $0)
Tonga	10 (5, 14)	2-yearly PWID testing + vaccination	6-monthly PWID testing	87 (79, 93)	74 (61, 85)	$214.28	$0 ($0, $0)	$0 ($0, $0)	$0 ($0, $0)

** Due to the high prevalence and incidence / reinfection rates among PWID, in scenarios without a vaccine even 6-monthly testing of PWID resulted in increased *cumulative* incidence. Therefore in the absence of other prevention measures, vaccine strategies among PWID were always non-dominated.

REFERENCES

1. Degenhardt L, Peacock A, Colledge S, et al. Global prevalence of injecting drug use and sociodemographic characteristics and prevalence of HIV, HBV, and HCV in people who inject drugs: a multistage systematic review. *The Lancet Global Health* 2017; **5**(12): e1192-e207.
2. Blach S, Zeuzem S, Manns M, et al. Global prevalence and genotype distribution of hepatitis C virus infection in 2015: a modelling study. *The Lancet Gastroenterology & Hepatology* 2017; **2**(3): 161-76.
3. Gower E, Estes C, Blach S, Razavi-Shearer K, Razavi H. Global epidemiology and genotype distribution of the hepatitis C virus infection. *J Hepatol* 2014; **61**(1 Suppl): S45-57.
4. Centre for Disease Analysis. 12 countries worldwide on track to eliminate hepatitis C infection by 2030. http://cdafound.org/just-12-countries-worldwide-on-track-to-eliminate-hepatitis-c-infection-by-2030-with-united-kingdom-italy-and-spain-among-those-joining-the-list/. 2018.
5. Scott N, Ólafsson S, Gottfreðsson M, et al. Modelling the elimination of hepatitis C as a public health threat in Iceland: a goal attainable by 2020. *Journal of hepatology* 2018; **68**(5): 932-9.
6. United Nations Department of Economic and Social Affairs Population Division. World Population Prospects: The 2015 Revision, DVD Edition. 2015.
7. World Health Organization. Global Hepatitis Report 2017. Geneva. Licence: CC BY-NC-SA 3.0 IGO. 2017.
8. The World Bank. GDP per capita. Available from: https://data.worldbank.org/indicator/NY.GDP.PCAP.CD?name_desc=false. 2016.
9. Guimarães ML, Marques BCL, Bertoni N, et al. Assessing the HIV-1 epidemic in Brazilian drug users: a molecular epidemiology approach. *PloS one* 2015; **10**(11): e0141372.
10. Oliveira MdLA, Yoshida CFT, Telles P, et al. Trends in HCV prevalence, risk factors and distribution of viral genotypes in injecting drug users: findings from two cross-sectional studies. *Epidemiology & Infection* 2009; **137**(7): 970-9.
11. Silva MBS, Andrade TM, Silva LK, et al. Prevalence and genotypes of hepatitis C virus among injecting drug users from Salvador-BA, Brazil. *Memórias do Instituto Oswaldo Cruz* 2010; **105**(3): 299-303.
12. Ministério da Saúde SdVeS. Boletim Epidemiológico Hepatites Virais. Brasília: Volume 49, N° 31. 2018.
13. Mathers BM, Degenhardt L, Bucello C, Lemon J, Wiessing L, Hickman M. Mortality among people who inject drugs: a systematic review and meta-analysis. *Bulletin of the World Health Organization* 2013; **91**(2): 102-23.
